# Integrated transcriptome and single-cell RNA sequencing identifies small GTPase-associated biomarkers in ulcerative colitis

**DOI:** 10.3389/fimmu.2026.1782885

**Published:** 2026-04-22

**Authors:** Meilin Chen, Weiguo Dong

**Affiliations:** 1 Department of Gastroenterology, Renmin Hospital of Wuhan University, Wuhan, China; 2 Key Laboratory of Hubei Province for Digestive System Disease, Renmin Hospital of Wuhan University, Wuhan, China

**Keywords:** ulcerative colitis, small GTPases, biomarkers, bioinformatics analysis, machine learning

## Abstract

**Background:**

Ulcerative colitis (UC) is a chronic, multifactorial inflammatory bowel disease. The involvement of small GTPase-related genes (SGRGs) in UC remains poorly defined. This study aims to identify SGRGs associated with UC and elucidate the molecular mechanisms by which they regulate the pathological progression of UC.

**Methods:**

Multiple transcriptomic datasets were integrated to identify candidate SGRGs in UC. Four machine learning algorithms were utilized for biomarker screening, followed by the construction of a diagnostic nomogram. The robustness of the identified biomarkers and the nomogram was rigorously evaluated through external validation in multiple independent cohorts. Furthermore, the associations between biomarkers and clinical severity, as well as their capacity for differential diagnosis (UC vs. Crohn’s disease (CD)), were assessed. Functional enrichment, immune infiltration, and cross-dataset single-cell RNA sequencing (scRNA-seq) were employed for in-depth mechanistic investigation. *In vivo* experiments validated core gene expression.

**Results:**

Three biomarkers (*ARHGEF3*, *S100A8*, and *RHOU*) were successfully identified and validated across multiple independent cohorts. The nomogram constructed based on these biomarkers exhibited excellent diagnostic performance (AUC = 0.991 in the training set, 0.938 and 0.968 in the external validation cohort). Notably, the expression levels of these biomarkers significantly correlated with clinical severity and pathological mucosal states, demonstrating their capacity to reflect disease activity and monitor progression. Furthermore, among the identified biomarkers, *ARHGEF3* significantly differentiated UC from CD (p < 0.05), while all three markers exhibited discriminative potential with AUC values exceeding 0.6. Functional annotation illustrated that they were commonly enriched within pathways encompassing “Tight junction” and “Leukocyte transendothelial migration”. Immune infiltration assessment demonstrated 27 differentially expressed immune cells (DEICs) among the UC and control groups. Cross-dataset scRNA-seq analysis further confirmed that macrophages were the key cells, exhibiting significant metabolic reprogramming and functional heterogeneity in UC. *In vivo* experiments confirmed the expression of above biomarkers in UC.

**Conclusion:**

This study systematically identifies *ARHGEF3*, *S100A8*, and *RHOU* as novel UC diagnostic biomarkers, implicating them in disease progression via immune regulation and macrophage function. These findings provide a new basis for precise diagnosis and targeted therapy.

## 1 Introduction

Ulcerative colitis (UC) constitutes a chronic, immune-driven inflammatory bowel disease (IBD) impacting the colon and rectum, primarily characterized by bloody diarrhea, abdominal pain, and weight loss, and it is a lifelong inflammatory disease (1–3). As the disease progresses, the risk of colorectal cancer in UC patients increases significantly (4, 5). In recent decades, incidence rates and prevalence of UC have been on the rise worldwide, especially in industrialized countries, where it has become an important public health issue (6). While the etiology of UC remains incompletely clarified, numerous studies indicate that it is strongly linked to genetic susceptibility, immune abnormalities, intestinal microbiota, and environmental factors (7). Despite the emergence of new drugs such as biologics that have changed the treatment paradigm of UC, the failure rate of drug treatment remains high (8). These clinical challenges highlight the urgency of deepening insight into the pathogenesis of UC, and finding novel biomarkers and therapeutic targets.

Small GTPases are a class of GTP-binding proteins that act as molecular switches playing a pivotal role in numerous cellular signaling pathways (9). Among them, small GTPases of the Ras homology (Rho) family (including Rac1, RhoA, and Cdc42) are important actin cytoskeleton regulators, playing a crucial role in cell spreading, migration, and growth (10). Recent studies have gradually revealed the special position of small GTPases in intestinal inflammation and the pathogenesis of UC. For instance, bacterial toxin (such as CNF1 toxin produced by enteropathogenic *Escherichia coli*) can irreversibly activate the Rac1 signaling pathway by specifically deamidating glutamine 61 of Rac1 small GTPase into glutamic acid (11). This post-translational modification of Rho protein abrogates the hydrolysis of GTP into GDP and the sustained GTP-loading of Rac1 Q61E sensitizes it to ubiquitin-mediated proteasomal degradation catalyzed by the HACE1 E3 ligase rate-limiting factor, which eventually leads to intracellular Rac1 depletion and disruption of cellular homeostasis (11). This mechanism not only reveals a new aspect of pathogen-host interaction but also provides a new perspective for understanding epithelial barrier dysfunction in UC.

Compared to traditional sequencing technologies, single-cell RNA sequencing (scRNA-seq) can disclose heterogeneity among cells within tissues, identify rare cell populations, and trace the trajectory of cell differentiation, providing a novel perspective for understanding the complex immune microenvironment of UC (12). In current UC research, single-cell technology has been successfully applied to depict disease-specific cellular maps (13). By comparing the inflammatory and non-inflammatory intestinal mucosa, researchers have discovered changes in the proportion and functional status of specific immune cell subsets in UC. For example, a previous study has shown that the colonic mucosa of UC patients often exhibits redistribution of macrophage subsets, with significant differences in metabolic activity, inflammatory factor production, and tissue repair capabilities among different subsets (14). These changes are closely linked to the severity and treatment response of UC and may serve as potential sources of biomarkers.

In the present research, small GTPase-related genes (SGRGs) in UC were systematically identified through the integration of multiple transcriptomic datasets. Biomarkers were subsequently screened using machine learning algorithms and rigorously validated across independent external cohorts to ensure their robustness. Furthermore, the clinical significance of these biomarkers in relation to disease severity and their capacity for differential diagnosis from Crohn’s disease (CD) were evaluated. Utilizing cross-dataset scRNA-seq analysis, the cell-type-specific regulatory mechanisms and key cellular niches involved in UC progression were explored. Finally, the results of bioinformatics analysis were validated at both mRNA and protein levels through the establishment of a murine colitis model. In conclusion, these findings provide novel diagnostic biomarkers for UC and offer new insights into clinical monitoring and the immune-regulatory mechanisms underlying the disease.

## 2 Materials and methods

### 2.1 Data source

The GSE87466 dataset was retrieved from the Gene Expression Omnibus (GEO, https://www.ncbi.nlm.nih.gov/gds) as the training set (platform: GPL13158), which included 87 UC patients and 21 normal colonic mucosa tissue samples. The GSE75214 dataset, comprising 97 UC patients and 11 normal colonic mucosa tissue samples, was used as the validation set (platform: GPL6244). The GSE231993 single-cell dataset contained 4 UC patients and 4 normal colonic mucosa tissue samples (platform: GPL18573) (15). Additionally, 733 SGRGs were sourced from the Molecular Signatures Database (MSigDB, https://www.gsea-msigdb.org/gsea/msigdb) (**Supplementary Table 1**). To further ensure the reliability and generalizability of the identified biomarkers, two additional independent transcriptomic datasets, GSE38713 and GSE107499, were retrieved from the GEO database for external validation. The GSE38713 dataset, based on the GPL570 platform, included 30 UC patients and 13 normal colonic mucosa tissue samples. The GSE107499 dataset, based on the GPL15207 platform, comprised 75 UC patients and 44 normal colonic mucosa tissue samples. These datasets were used to validate the expression trends and diagnostic value of the biomarkers. The GSE11223 dataset (platform: GPL1708) contained 202 samples. After excluding 12 terminal ileum samples and 67 control colon samples, the remaining 123 UC colon samples with available clinical scores were stratified by disease severity into a Remission/Mild group (n = 71), a Moderate group (n = 38), and a Severe group (n = 14) for investigating the relationship between biomarkers and disease progression.

Additionally, to assess phenotypic specificity, the CD samples (n = 8) from the GSE75214 dataset were re-included for comparative analysis between UC, CD, and healthy controls.

### 2.2 Identification of candidate genes, functional enrichment, and establishment of protein-protein interaction network

To screen for differentially expressed genes (DEGs) among the UC group and control group, differential expression analysis was carried out on the GSE87466 training set via the limma package (v3.54.2) (16), adopting the cutoff criteria of |log_2_fold change (FC)| > 1 and adjusted p (p.adj)-value < 0.05. A volcano plot was plotted via the ggplot2 package (v3.5.1) (17), with the top 10 significantly upregulated and downregulated genes ranked by |log_2_FC| labeled. Meanwhile, a heatmap of these 20 DEGs was produced by means of the pheatmap package (v1.46.0) (18). Finally, candidate genes (CGs) were obtained via extracting the intersection of DEGs and SGRGs with the ggvenn package (v0.1.9) (19).

Subsequently, Gene Ontology (GO) (p.adj < 0.05) and Kyoto Encyclopedia of Genes and Genomes (KEGG) (p < 0.05) enrichment analyses were conducted on CGs via the clusterProfiler package (v4.7.1.3) (20). GO analysis encompassed three dimensions: biological processes, molecular functions, and cellular components. The top 5 most significant pathways in each aspect, ranked by count, were displayed. To explore the network interaction relationships of CGs at the protein level, protein-protein interaction (PPI) analysis was undertaken by means of the Search Tool for the Retrieval of Interacting Genes/Proteins (STRING) database (https://string-db.org/) (confidence > 0.4, node connectivity ≥ 1). Finally, the PPI network was constructed using the circlize package (v0.4.15) (21). To identify the hub nodes within the PPI network, the Degree centrality algorithm was utilized. Degree centrality is defined as the total number of direct interactions (edges) linked to a specific node, reflecting the connection density and local regulatory importance of genes within the network. The genes with the highest Degree scores were designated as the core regulators of the molecular regulatory network.

### 2.3 Identification of biomarkers

To screen for biomarkers, least absolute shrinkage and selection operator (LASSO) regression analysis was first conducted on the CGs via the glmnet package (v4.1.4) (22) based on all samples in the training set. Following 10-fold cross-validation, feature genes 1 (FGs1) were selected following the criterion of minimizing the model error rate. Subsequently, support vector machine-recursive feature elimination (SVM-RFE) analysis was conducted using the caret package (v6.0.93) (23). After 10-fold cross-validation, the gene combination where the accuracy first reached the peak was chosen as feature genes 2 (FGs2). A random forest (RF) classification model was constructed by employing the randomForest package (v4.7.1.1) (24) with ntree = 31, and the top 5 genes sorted by importance were designated as feature genes 3 (FGs3). Boruta analysis was carried out using the Boruta package (v8.0.0) (25) in which the upper limit of iteration set to 1000, resulting in the screening of feature genes 4 (FGs4). Finally, the ggvenn package (v0.1.9) was used to take the intersection of FGs1, FGs2, FGs3, and FGs4 to obtain candidate biomarkers (CBs). Further, Wilcoxon test was utilized to analyze discrepancies in expression of CBs among the UC group and control group across the two datasets. CBs with notable expression differences (p < 0.05) and concordant expression trends across both datasets were recognized as key candidate biomarkers. Ultimately, receiver operating characteristic (ROC) curve analysis was implemented with respect to the key CBs in both datasets via the pROC package (v1.18.0) (26), and area under the curve (AUC) values were calculated. Key CBs with AUC values greater than 0.7 and not equal to 1 in both datasets were identified as the final biomarkers.

### 2.4 Establishment and assessment of the nomogram

To evaluate the predictive efficacy of these biomarkers for UC, this study established a nomogram model using the rms package (v6.5.0) based on the GSE87466 dataset (27). The predictive performance and clinical utility of the nomogram were first evaluated within the training cohort and subsequently validated using the independent GSE38713 dataset and GSE107499 dataset as an external validation cohort. The probability of UC occurrence in individuals was predicted using the total score of the biomarkers. The nomogram model was further validated through multiple methods: the pROC package (v1.18.0) was utilized to generate the ROC curve and calculate the AUC value; the rms package (v6.5.0) was employed to generate a calibration curve, which evaluated the correspondence between the forecasted probabilities and actual occurrence rates (28); decision curve analysis (DCA) was performed using the rmda package (v1.6) to assess the clinical net advantage of the model at distinct threshold probabilities (29).

### 2.5 Gene set enrichment analysis

The reference gene set (c2.cp.kegg_legacy.v2025.1.Hs.symbols.gmt) was obtained from the MSigDB. Based on all samples in the GSE87466 dataset, the psych package (v2.2.9) (30) was utilized to compute Spearman’s correlation coefficients between the biomarkers and all other genes, and the genes were sorted in descending order of these correlation coefficients. Gene set enrichment analysis (GSEA) was conducted via the clusterProfiler package (v4.7.1.3) to explore the signaling pathways associated with the expression patterns of these biomarkers in UC (with criteria: p.adj < 0.05). The top 5 most significant pathways ranked by p-value were selected for visualization. The ggvenn package (v0.1.9) was used to take the intersection of the enriched pathways of the biomarkers to obtain common pathways.

### 2.6 Immune infiltration analysis

To estimate the relative infiltration enrichment scores of 28 immune cell subtypes, the ssGSEA algorithm implemented in the GSVA package (v1.46.0) was applied to the GSE87466 bulk transcriptomic dataset (31). Wilcoxon test was performed to compare the discrepancies in immune cell infiltration among the UC group and control group, and immune cells with p < 0.05 were classified as DEICs. Additionally, the psych package (v2.2.9) was applied to carry out Spearman’s correlation analysis between DEICs, as well as between DEICs and biomarkers (with criteria: |cor| > 0.3 and p < 0.05), and the pheatmap package (v1.46.0) was employed to generate correlation heatmaps.

### 2.7 scRNA-seq analysis

ScRNA-seq data from two independent datasets, GSE231993 and GSE125527 (32), were processed and analyzed using the Seurat package (v5.0.1) (33). Uniform filtering criteria were applied to both datasets: genes per cell ranging from 200 to 3000; genes with an expression level ≤ 15000 and detected in at least 3 cells retained; and the proportion of mitochondrial genes < 15%. The distributions of nCount_RNA, nFeature_RNA, and percent.mt before and after quality control were presented. After data normalization, the vst method was used to screen the leading 2000 highly variable genes (HVGs), and the highest-ranked 10 HVGs were annotated. To eliminate technical batch effects, we first performed principal component analysis (PCA) on the top 2000 HVGs using the RunPCA function, and the initial batch-wise distribution of samples was visualized in the PCA space via the DimPlot function to assess potential technical variations. Subsequently, we applied the Harmony algorithm to integrate and correct the data, minimizing technical noise. We evaluated the statistical significance of the principal components using the JackStraw and ElbowPlot functions, and finally determined the principal components for subsequent analysis (p < 0.05). Unsupervised clustering of cells was conducted via the FindNeighbors and FindClusters functions (resolution = 0.4 for GSE231993 and 0.5 for GSE125527), and the results were displayed through uniform manifold approximation and projection (UMAP). Next, marker genes from the literature (34) were compared with the specifically highly expressed genes of each cell cluster retrieved from the CellMarker database, so as to annotate the cells in each cluster. The annotated cell clusters were visualized using UMAP graphs, and bubble plots were generated to depict the expression patterns of the marker genes. To further identify key cells playing roles in UC, the proportional distribution of each cell type in normal and UC samples was presented, and the Chi-square test was used to assess the significance of intergroup differences. Cells with significantly different expression of biomarkers and high proportions in both groups were selected as key cells.

### 2.8 Cell-cell communication and metabolic pathway analysis

To analyze cell-cell interactions, cell communication analysis was conducted via the CellChat package (v1.6.1) in both UC and normal samples from the GSE231993 dataset, generating graphs of the number and intensity of intercellular interactions (35). Subsequently, the scMetabolism package (v0.2.1) (36) was utilized to perform metabolic pathway analysis on each cell type, aiming to identify the highly active metabolic pathways in each cell.

### 2.9 Functional enrichment and pseudotime trajectory analysis of key cells

First, clustering was performed on the key cells, and cell functional enrichment analysis was carried out via the ReactomeGSA package (v1.12.0) (37). Subsequently, the monocle package (v2.26.0) (35) was utilized to perform pseudotime trajectory analysis on the key cells. A single-cell trajectory graph was constructed via the DDRTree function to explore the developmental and differentiation trajectories of the key cells. Additionally, the pseudotime expression trajectory plots of the biomarkers in the cells were presented.

### 2.10 Preparation of experimental animals

We selected aged 6 weeks C57BL/6J wild-type (WT) mice (male) provided by GemPharmatech Co., Ltd (Nanjing, China), and raised them under specific pathogen-free conditions with appropriate temperature, humidity and sufficient autoclaved food. After a 1-week of acclimatization period, the mice were randomly divided into the control group and the disease group of 6 mice each, and the disease model was constructed by drinking sterile water containing 3% (w/v) dextran sulfate sodium (DSS) (MP Biomedicals, Irvine, CA, USA) for 7 consecutive days. Then we observed and recorded the general condition, body weight, stool consistency and hematochezia of mice in each group every day. The mice were euthanized on the eighth day, the colon length was measured quickly, and the colon specimens were embedded in paraffin or stored in liquid nitrogen for subsequent analysis. Histological scores and disease activity index (DAI) were evaluated according to our previous report (7). All animal experiments were conducted in compliance with the “Guidelines for the Care and Use of Laboratory Animals” and approved by the Institutional Animal Care and Use Committee of Renmin Hospital of Wuhan University (Approval number: WDRM20231004B).

### 2.11 RNA extraction and quantitative RT-PCR

Total RNA was extracted from mice tissues via the TRIzol reagent (Invitrogen, Waltham, MA, USA) and reverse transcribed using the PrimeScript RT reagent Kit with gDNA Eraser (TaKaRa, Shiga, Japan; Cat. No. RR047A). TB Green (Takara, Japan; Cat. No. RR820A) was used to conduct quantitative real-time polymerase chain reaction (qRT-PCR) on a Bio-Rad CFX PCR instrument. The relative expression level of each gene was calculated according to the 2^-ΔΔCt^ method. All primers used for amplification are listed as following:

*ARHGEF3*, forward: CTTCAGCAACCACGAGAGAGT, reverse: GAAGGTGTCGTTGGCTTGTA.

*S100A8*, forward: TCACCATGCCCTCTACAAGAA, reverse: TTATCACCATCGCAAGGAACT.

*RHOU*, forward: GAGAAGCCGGTGCCTGAAGA, reverse: CTTTGGCTGTAGCTGGGAGT.

*GAPDH*, forward: TCCCACTCTTCCACCTTCGA, reverse: CAGGAAATGAGCTTGACAAAGTTG.

### 2.12 Western blot analysis

Total protein was extracted from colonic tissues using radioimmunoprecipitation assay (RIPA) buffer (Sigma-Aldrich, St Louis, MO, USA) supplemented with protease and phosphatase inhibitors. Protein concentrations were determined using the BCA Protein Assay Kit (Thermo Fisher Scientific, Waltham, MA, USA). Equal amounts of proteins were separated by 10% sodium dodecyl sulfate-polyacrylamide gel electrophoresis (SDS-PAGE) and transferred onto polyvinylidene fluoride (PVDF) membranes.

The antibodies used were as follows: ARHGEF3 (#DF4434; Affinity Biosciences, Cincinnati, OH, USA), S100A8 (#15792-1-AP; Proteintech, Wuhan, China), and RHOU (#PA5-95893; Thermo Fisher Scientific), with β-actin (#66009-1-Ig; Proteintech) serving as the internal control. Following incubation with horseradish peroxidase-conjugated secondary antibodies, protein bands were visualized using an enhanced chemiluminescence (ECL) system.

### 2.13 Statistical analysis

All statistical analyses and visualization were performed using R software (v4.2.2). The Wilcoxon rank-sum test was employed to determine intergroup differences between two independent groups (e.g., UC vs. Control, or UC vs. CD). For comparisons involving multiple clinical stages (e.g., Remission/Mild, Moderate, and Severe stages), pairwise Wilcoxon tests were utilized. For the evaluation of cell type proportion differences in scRNA-seq datasets, the Chi-square test was applied. The diagnostic efficacy of individual biomarkers and the nomogram was assessed via ROC curve analysis, with the AUC calculated using the pROC package. A p-value < 0.05 was considered statistically significant.

## 3 Results

### 3.1 Screening of CGs, functional enrichment analysis, and construction of interaction networks in UC

**Figure 1:**
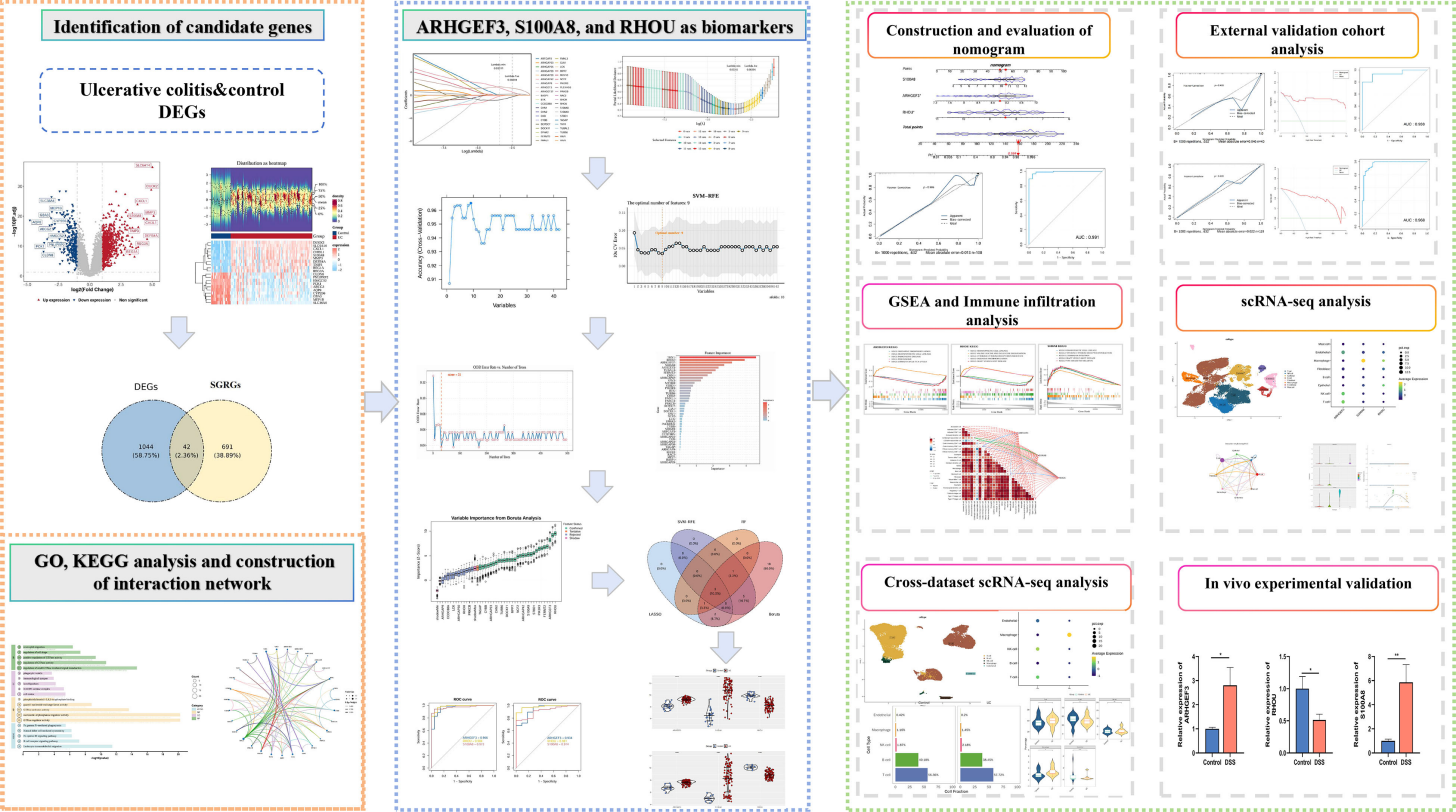
The workflow of this study.

**Figure 2:**
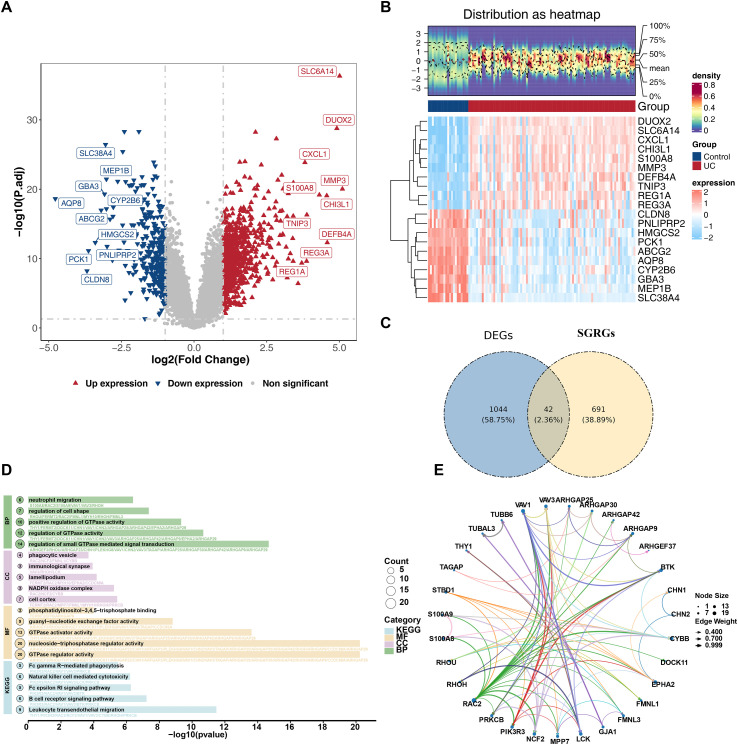
Screening of candidate genes (CGs), functional enrichment analysis, and construction of interaction networks in ulcerative colitis (UC). **(A)** Volcano plot of differentially expressed genes (DEGs) between UC samples and healthy controls. The y-axis represents -log10[adjusted p (p.adj)-value], and the x-axis denotes log2fold change. Each dot in the plot corresponds to a gene: blue dots indicate downregulated genes, red dots represent upregulated genes, and gray dots signify non-significantly DEGs. **(B)** Heatmap of DEGs expression. The heatmap consists of two parts. The upper part displays a density heatmap of the top 20 DEGs (10 upregulated and 10 downregulated), showing the mean line and five quantile lines. The lower part is an expression heatmap, where each row represents a gene and each column represents a sample. In the expression heatmap, color intensity reflects gene expression levels, with red indicating high expression and blue indicating low expression. **(C)** Venn diagram of overlapping genes between DEGs and small GTPase-related genes (SGRGs) to identify CGs. **(D)** Gene Ontology (GO) enrichment analysis (p.adj < 0.05) and Kyoto Encyclopedia of Genes and Genomes (KEGG) pathway enrichment analysis (p < 0.05). **(E)** Protein-protein interaction (PPI) network of CGs.

The workflow was illustrated in **Figure 1**. In this study, altogether 1086 DEGs were identified in UC samples by way of differential expression analysis, including 750 upregulated genes and 336 downregulated genes (**Supplementary Table 2**). Among these, genes such as *MMP3*, *SLC6A14*, and *DUOX2* were significantly upregulated, while genes including *AQP8*, *CLDN8*, and *PCK1* were remarkably downregulated, indicating their potential critical roles in the pathological process of UC (**Figures 2A, B**). To further focus on the mechanism of small GTPases in UC, the intersection of DEGs and SGRGs was taken to screen 42 CGs, such as *ARHGEF3*, *S100A8*, and *RHOU* (**Figure 2C**; **Supplementary Table 3**). GO enrichment analysis identified 191 significant pathways (p.adj < 0.05): 140 enriched BPs, including “Regulation of small GTPase mediated signal transduction” and “Regulation of GTPase activity”; 16 CCs, such as “Cell cortex” and “NADPH oxidase complex”; and 35 MFs, including “GTPase regulator activity” and “Nucleoside-triphosphatase regulator activity” (**Figure 2D**; **Supplementary Table 4**). KEGG analysis yielded 65 significant pathways (p < 0.05), including “Leukocyte transendothelial migration” and “B cell receptor signaling pathway” (**Figure 2D**; **Supplementary Table 5**). Additionally, PPI network analysis revealed close interactions among 31 CGs, forming 83 associations in total. Based on the Degree centrality metric, RAC2, NCF2, and LCK served as hub nodes in the network with the highest connectivity, demonstrating that they might serve as core mediators in the UC-related molecular regulatory network (**Figure 2E**; **Supplementary Table 6**).

### 3.2 *ARHGEF3*, *S100A8*, and *RHOU* as biomarkers

**Figure 3:**
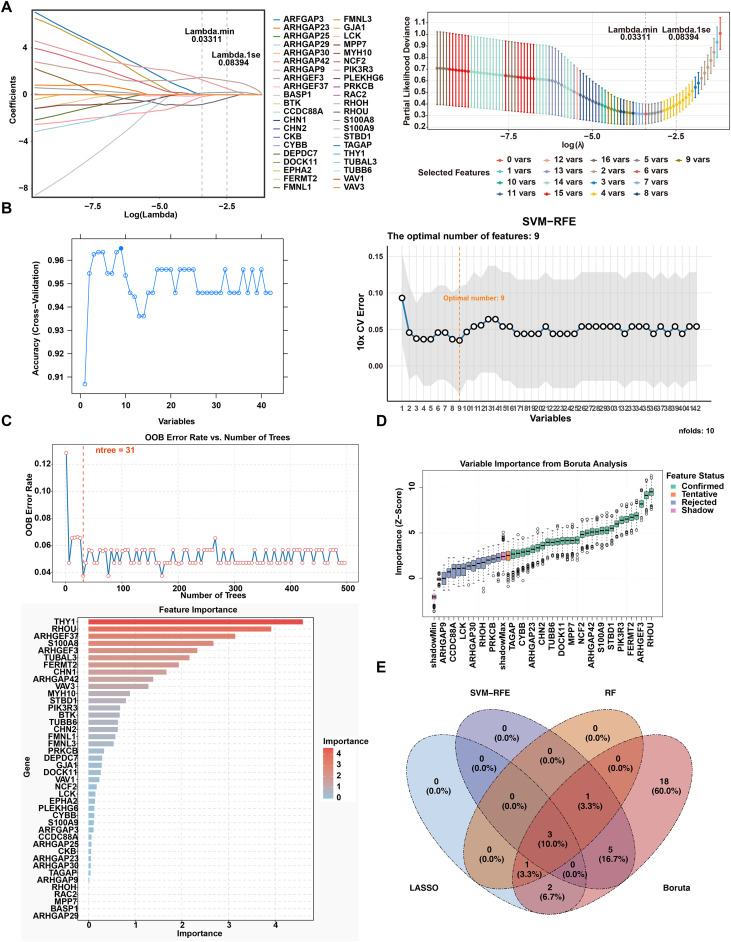
*ARHGEF3*, *S100A8*, and *RHOU* as biomarkers. **(A)** Feature genes 1 (FGs1) screened by the least absolute shrinkage and selection operator (LASSO) regression. The left panel shows the LASSO coefficient profile, and the right panel presents cross-validation for parameter adjustment in LASSO analysis. The x-axis represents the logarithm of lambdas, and the y-axis denotes variable coefficients (each line corresponds to a gene). As lambda increases, gene coefficients tend toward 0. At the optimal lambda (selected as the minimum lambda value), genes with coefficients of 0 are eliminated. **(B)** Feature genes 2 (FGs2) screened by the support vector machine-recursive feature elimination (SVM-RFE) algorithm. **(C)** Feature genes 3 (FGs3) screened by the random forest (RF) algorithm. The upper subplot shows the out-of-bag (OOB) error rate of the model at different ntree values (blue line = error rate trend, red dots = actual error rates). The lower subplot presents the importance ranking of feature genes identified by RF: the x-axis indicates feature importance values, the y-axis lists core genes, and the colors gradient from blue to red represent increasing importance. **(D)** Feature genes 4 (FGs4) identified by the Boruta algorithm. **(E)** Venn diagram of overlapping feature genes.

**Figure 4:**
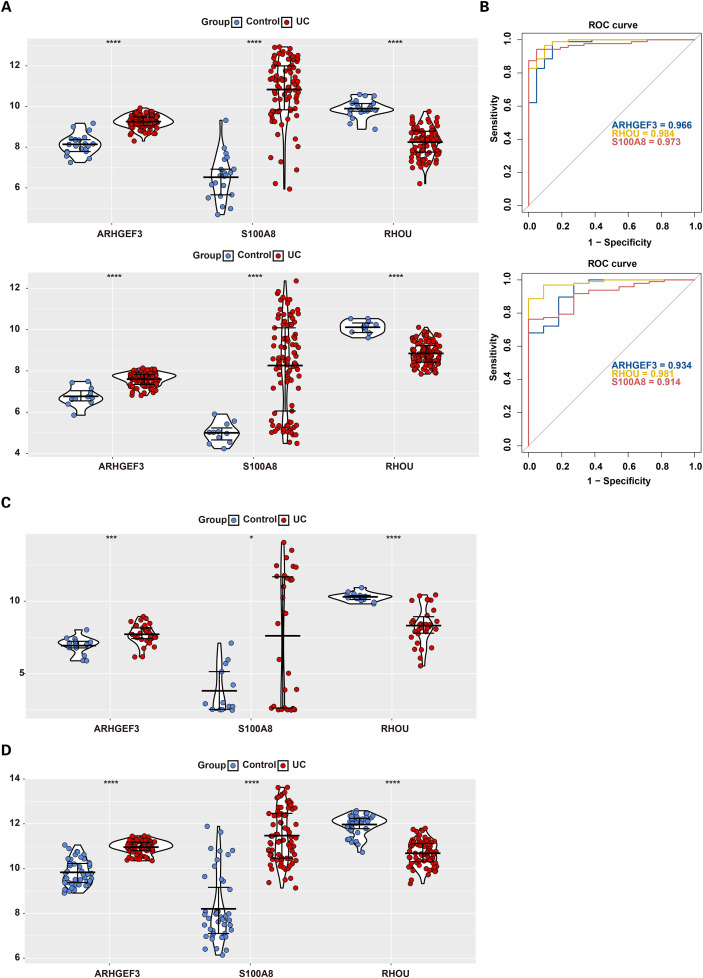
Validation of *ARHGEF3*, *S100A8*, and *RHOU* across multiple independent datasets. **(A)** Comparison of candidate biomarkers (CBs) expression levels between the training and validation sets. The y-axis represents gene expression levels, and the x-axis distinguishes between control and ulcerative colitis (UC) groups. **(B)** Receiver operating characteristic (ROC) curves of CBs in the training and validation sets. The x-axis denotes the false positive rate (1-specificity), the y-axis represents the true positive rate (sensitivity), and the area under the curve (AUC) indicates the area under the ROC curve. **(C, D)** External validation of *ARHGEF3*, *S100A8*, and *RHOU* expression in independent cohorts. Significance markers: *p < 0.05, ***p < 0.001, ****p < 0.0001.

LASSO regression analysis identified 6 FGs1, including *ARHGEF3*, *S100A8*, *RHOU*, *PIK3R3*, *ARHGEF37*, and *ARHGAP42* (**Figure 3A**) (Lambda min = 0.03311). SVM-RFE analysis screened 9 FGs2, such as *RHOU*, *TUBAL3*, *ARHGEF3*, *FERMT2*, *CHN1*, *S100A8*, *VAV3*, *THY1*, and *STBD1* (**Figure 3B**). The RF model achieved optimal performance when ntree = 31. Based on the ranking of gene importance, 5 FGs3 were obtained, namely *THY1*, *RHOU*, *ARHGEF37*, *S100A8*, and *ARHGEF3* (**Figure 3C**). Boruta analysis confirmed 30 FGs4, including *THY1*, *PIK3R3*, *STBD1*, etc. (**Figure 3D**; **Supplementary Table 7**). By taking the intersection of the above four sets of FGs, 3 common CBs were identified: *ARHGEF3*, *S100A8*, and *RHOU* (**Figure 3E**). Subsequently, the expression levels of these three genes were validated in both the training set and the validation set. The findings demonstrated that relative to the control group, *ARHGEF3* and *S100A8* were notably upregulated in the UC group, while *RHOU* was remarkably downregulated (**Figure 4A**). Additionally, their diagnostic efficacy, as indicated by the AUC values, were all greater than 0.7, demonstrating excellent discriminatory ability (**Figure 4B**). To ensure robustness, external validation was performed using the GSE38713 and GSE107499 datasets. Consistent with the initial findings, *ARHGEF3* and *S100A8* were significantly upregulated, while *RHOU* was notably downregulated in all UC samples (**Figures 4C, D**). Therefore, *ARHGEF3*, *S100A8*, and *RHOU* were ultimately confirmed as potential diagnostic biomarkers for UC.

**Figure 5:**
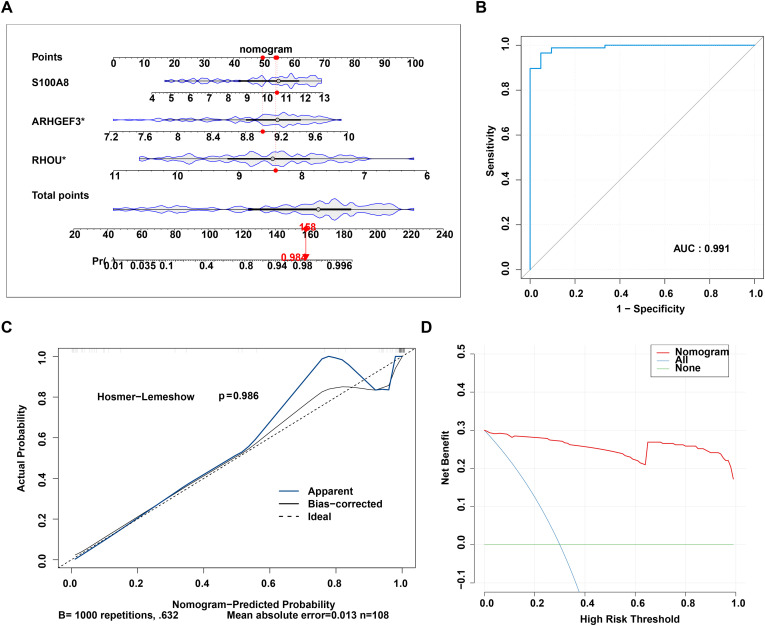
Construction and evaluation of the nomogram. **(A)** Nomogram. **(B)** Receiver operating characteristic (ROC) curve. **(C)** Calibration curve. **(D)** Decision curve.

A diagnostic nomogram was constructed based on the training set (GSE87466) to quantify the risk of UC (**Figure 5A**). In the training cohort, the model demonstrated superior diagnostic performance with an AUC of 0.991 (**Figure 5B**). Calibration analysis via the Hosmer-Lemeshow test (p = 0.986) validated the high predictive accuracy, while DCA confirmed the high clinical utility of the model (**Figures 5C, D**).

**Figure 6:**
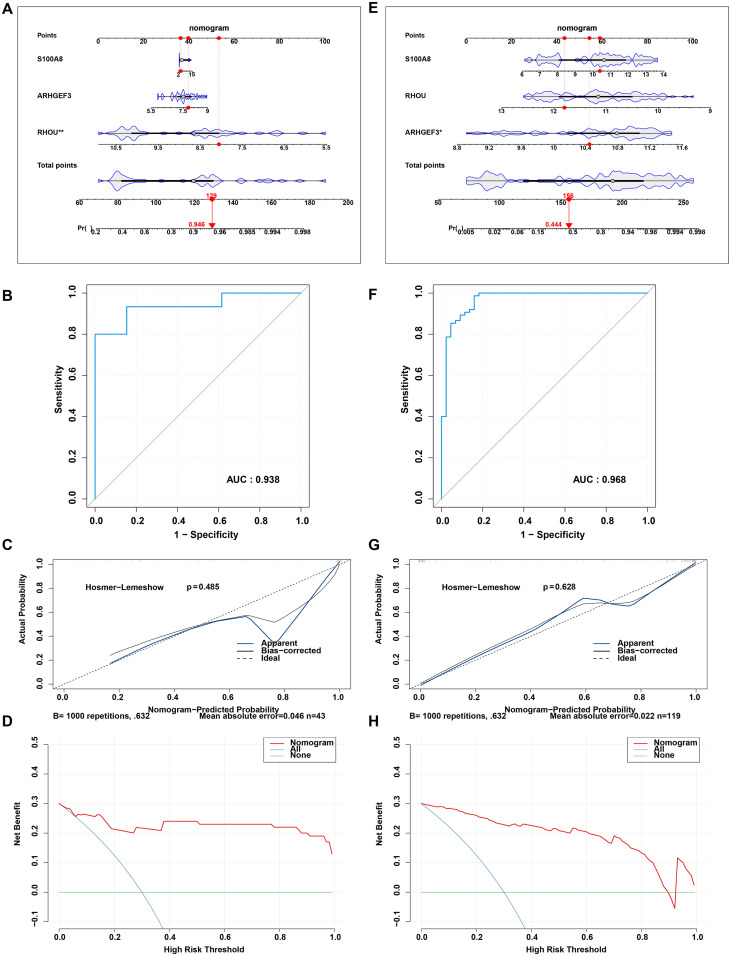
External validation of the diagnostic nomogram in two independent cohorts (GSE38713 and GSE107499). **(A–D)** Performance evaluation of the nomogram in the GSE38713 cohort. **(A)** Visualization of the diagnostic nomogram for ulcerative colitis (UC) risk prediction. **(B)** Receiver operating characteristic (ROC) curve analysis yielding an area under the curve (AUC) of 0.938. **(C)** Calibration curve showing the agreement between predicted and actual probabilities; the Hosmer-Lemeshow test yielding a p-value of 0.485. **(D)** Decision curve analysis (DCA) illustrating the clinical net benefit of the model. **(E–H)** Performance evaluation of the nomogram in the GSE107499 cohort. **(E)** Visualization of the diagnostic nomogram. **(F)** ROC curve analysis yielding an AUC of 0.968. **(G)** Calibration curve demonstrating high predictive accuracy, with a Hosmer-Lemeshow test p-value of 0.628. **(H)** DCA confirming the robust clinical utility of the nomogram across different independent populations.

To evaluate the generalizability of the nomogram, it was applied to the independent GSE38713 and GSE107499 datasets. In the GSE38713 cohort, the nomogram effectively quantified the risk of UC (**Figure 6A**), demonstrating excellent diagnostic performance with an AUC of 0.938 (**Figure 6B**). The predictive accuracy was validated through calibration analysis using the Hosmer-Lemeshow test (p = 0.485), while the high clinical utility of the model was confirmed by DCA (**Figures 6C, D**). Similarly, the model exhibited outstanding diagnostic performance in the GSE107499 dataset, yielding an AUC of 0.968 (**Figures 6E, F**). Calibration analysis via the Hosmer-Lemeshow test indicated a high degree of predictive accuracy (p = 0.628), and the clinical net benefit was further supported by DCA (**Figures 6G, H**).

### 3.3 Correlation of biomarkers with UC clinical severity and differential diagnosis from CD

**Figure 7:**
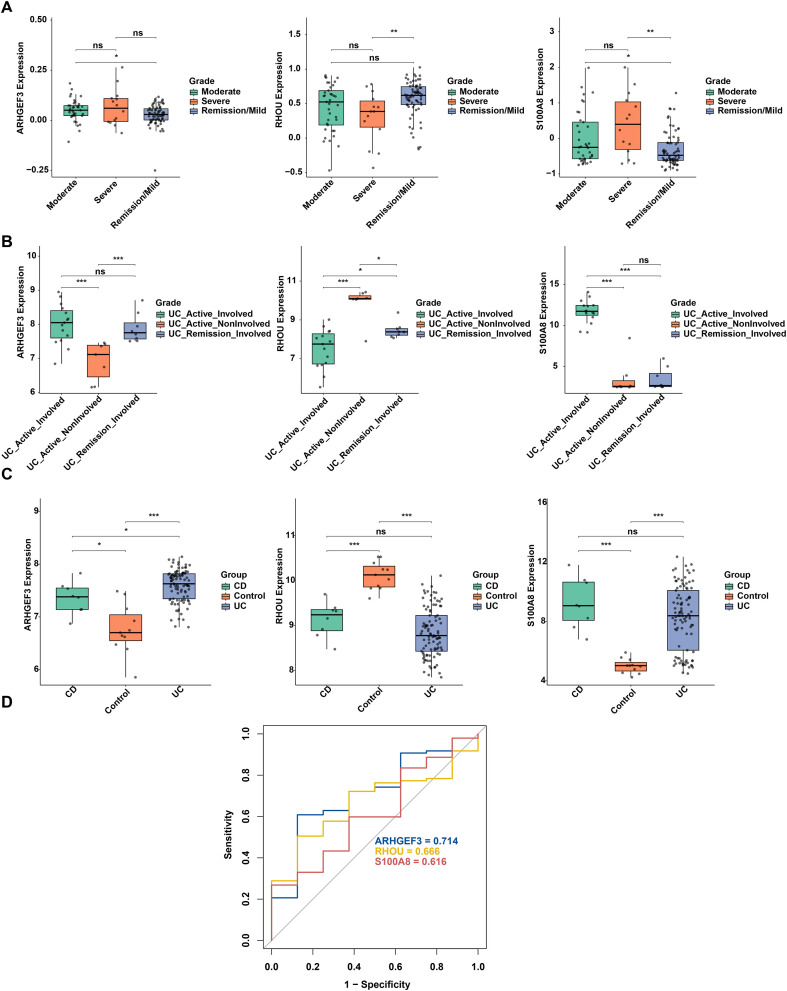
Expression profiles of candidate biomarkers in different clinical severity and colitis phenotypes of ulcerative colitis (UC). **(A)** Expression levels of *ARHGEF3*, *RHOU*, and *S100A8* in the GSE11223 cohort across different clinical severity grades (Remission/Mild, Moderate, and Severe). **(B)** Expression of the three biomarkers in the GSE38713 cohort across pathological subgroups, including UC_Active_Involved, UC_Active_NonInvolved, and UC_Remission_Involved groups. **(C)** Differential expression analysis of biomarkers in the GSE75214 dataset among healthy control (Control), UC and Crohn’s disease (CD) groups. **(D)** Receiver operating characteristic (ROC) curves for evaluating the performance of *ARHGEF3* (area under the curve (AUC) = 0.714), *RHOU* (AUC = 0.666), and *S100A8* (AUC = 0.616) in differentiating UC from CD. Significance markers: *p < 0.05, **p < 0.01, ***p < 0.001. ns, not significant.

To explore the association between three biomarkers and the clinical progression of UC, this study analyzed the expression of these biomarkers in 123 UC colon samples from the GSE11223 dataset, which included Remission/Mild, Moderate, and Severe disease subgroups. The analysis (**Figure 7A**) showed that the expression level of *S100A8* increased with disease severity and was significantly higher in both the Moderate and Severe groups than in the Remission/Mild group. The expression level of *ARHGEF3* differed significantly between the Remission/Mild group and the Moderate group, and was markedly upregulated in the Moderate group. In contrast, the expression level of *RHOU* differed significantly between the Remission/Mild group and the Severe group, and was significantly downregulated in the Severe group.

Further validation was conducted using the GSE38713 dataset across three distinct pathological states (**Figure 7B**). The results demonstrated that *ARHGEF3* expression was significantly higher in both the UC_Active_Involved and UC_Remission_Involved groups compared to the UC_Active_NonInvolved group; however, no significant difference was observed between the involved-active and remission states. *RHOU* exhibited significant expression disparities among all three pathological subtypes, with the highest abundance recorded in the UC_Active_NonInvolved group and the lowest in the UC_Active_Involved group. Conversely, the expression of *S100A8* was dramatically elevated in the UC_Active_Involved group compared to both the UC_Active_NonInvolved and UC_Remission_Involved groups, whereas no significant variation was found between the latter two groups.

To address phenotypic specificity, CD samples were re-included from the GSE75214 dataset. The analysis demonstrated that *ARHGEF3* exhibited significant discriminative capacity between UC and CD. ROC curve analysis revealed an AUC value > 0.7, confirming its potential as a phenotypic-specific diagnostic marker for UC rather than a general marker for intestinal inflammation. Although *S100A8* and *RHOU* showed no significant differences between UC and CD, their AUC values > 0.6 indicated certain predictive value for the differential diagnosis of UC/CD (**Figures 7C, D**). These findings indicate that *ARHGEF3* provides better discrimination between UC and CD patients.

### 3.4 Functional pathways of biomarkers

**Figure 8:**
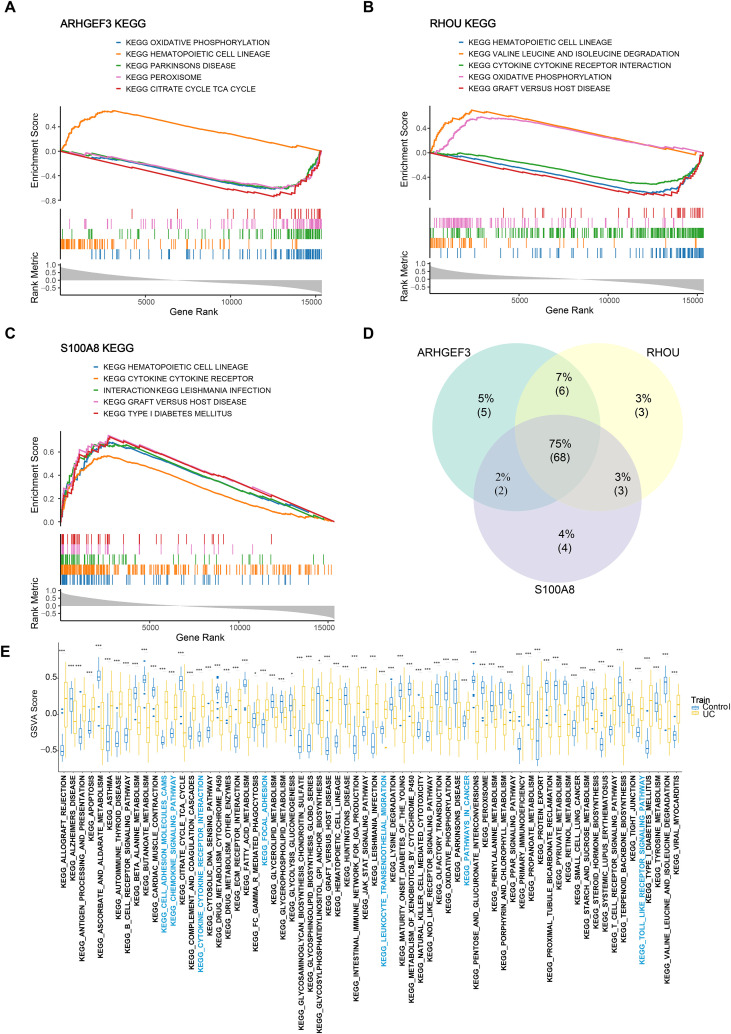
Functional enrichment analysis of biomarkers. **(A)** Gene set enrichment analysis (GSEA) results for *ARHGEF3*. **(B)** GSEA results for *RHOU*. **(C)** GSEA results for *S100A8*. **(D)** Venn diagram of pathway intersections among the three biomarkers. **(E)** Differences in common pathways between the disease and control groups. Significance markers: *p < 0.05, ***p < 0.001.

Prior to functional exploration, the fundamental biological attributes of the three SGRG biomarkers, including their subcellular localization and chromosomal mapping, were characterized (**Supplementary Figures 1A–D**). Subsequently, KEGG enrichment analysis was performed to elucidate the functional landscape of the three biomarkers (**Figures 8A–C**). Overlap analysis revealed that the biomarker-associated gene sets were co-enriched in 68 pathways, and the GSVA scores for these pathways differed significantly between the UC and control groups. Among these, the GSVA scores for pathways such as “Leukocyte transendothelial migration” and “Focal adhesion” were significantly higher than those in the control group, whereas the GSVA score for “Tight junction” was significantly lower than that in the control group (**Figures 8D, E**; **Supplementary Table 8**). The concerted dysregulation of these pathways collectively reflects the pathological features of impaired intestinal barrier function and inflammatory cell infiltration in UC.

### 3.5 Immune cell infiltration characteristics and biomarker correlation analysis

**Figure 9:**
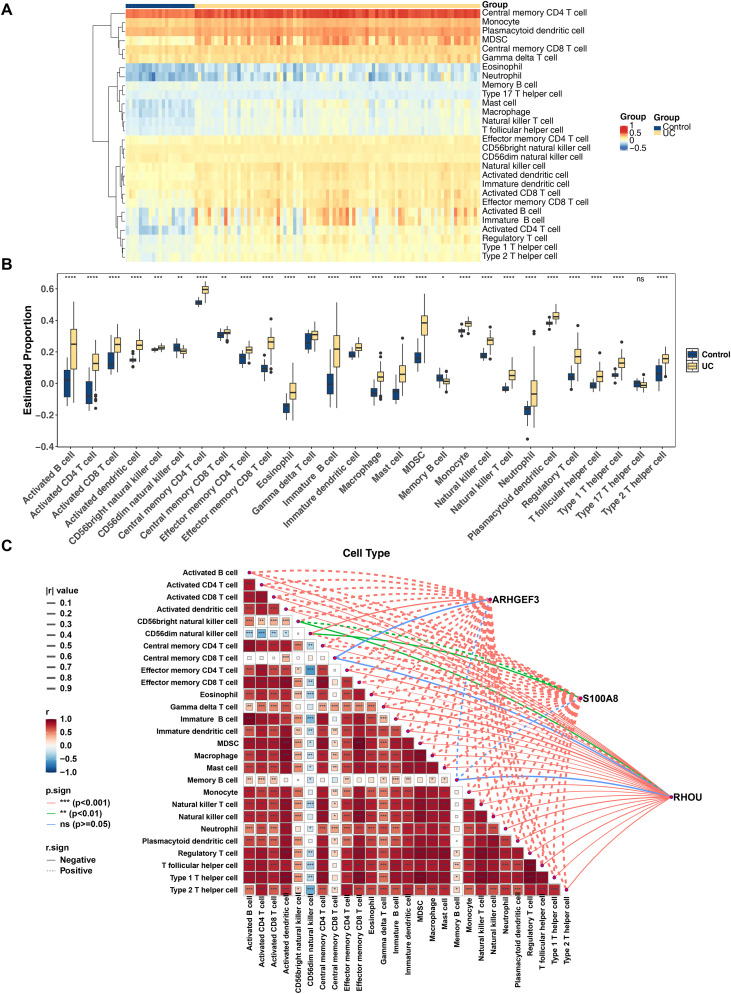
Immune cell infiltration analysis between ulcerative colitis (UC) and control groups. **(A)** Infiltration profiles of 28 immune cell types. Colors gradient from blue to orange indicate increasing infiltration levels; the x-axis represents samples from different groups, and the y-axis lists immune cell types. **(B)** Boxplots of differentially expressed immune cells (DEICs) between UC and normal groups. **(C)** The correlation between biomarkers and DEICs. Colors gradient from blue to red represent negative to positive correlations. Significance markers: *p < 0.05, **p < 0.01, ***p < 0.001, ****p < 0.0001. ns, not significant.

To probe changes in the immune microenvironment of UC, the infiltration enrichment scores of 28 immune cell subsets were evaluated in the GSE87466 dataset using bulk deconvolution analysis. The results showed that central memory CD4 T cells displayed elevated infiltration levels in most UC and control samples (**Figure 9A**). Further analysis confirmed that except for Type 17 T helper cells, the infiltration abundances of the other 27 immune cell types were significantly different (p < 0.05). Among these, CD56dim natural killer cells and memory B cells were significantly downregulated in the UC group, while the remaining 25 cell types were remarkably upregulated (**Figure 9B**). Correlation profiling of immune cells indicated a markedly positive association between MDSCs (Myeloid-Derived Suppressor Cells) and effector memory CD8 T cells (p < 0.001), and a strong negative correlation between CD56dim natural killer cells and effector memory CD4 T cells (p < 0.001). Additionally, correlation analysis between biomarker expression and immune cell infiltration indicated that *S100A8* had a significant positive correlation with activated dendritic cells (cor = 0.94, p < 0.001); *RHOU* showed the most pronounced inverse relationship with effector memory CD8 T cells (cor = -0.83, p < 0.001) (**Figure 9C**; **Supplementary Table 9**). These findings strongly suggest that *ARHGEF3*, *S100A8*, and *RHOU* may be deeply involved in the immune-inflammatory process of UC by regulating the recruitment or function of specific immune cell subsets, supplying fresh cellular evidence to comprehend the immunopathogenesis of UC.

### 3.6 Single-cell transcriptome analysis deciphers the UC cellular atlas and identifies macrophages as key cells

**Figure 10:**
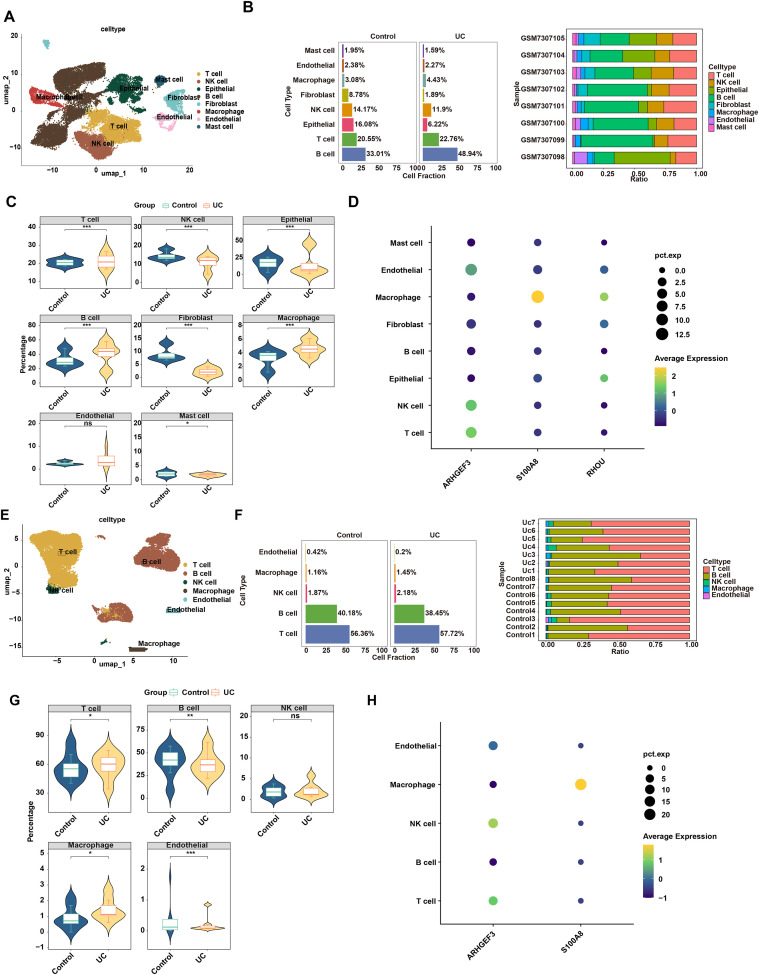
Cross-dataset single-cell transcriptomic landscape and validation of biomarker expression in macrophages. **(A–D)** Results from the GSE231993 training cohort. **(A)** Visualization of annotated cell clusters. **(B)** Stacked bar chart of cell proportions in the control and ulcerative colitis (UC) groups. **(C)** Differential cell composition between control and UC groups. **(D)** Dot plot of biomarker expression in various cell types (where dot size and yellow intensity correspond to expression abundance). **(E–H)** External validation using the GSE125527 cohort. **(E)** Visualization of annotated cell clusters. **(F)** Stacked bar chart of cell proportions in the control and UC groups. **(G)** Differential cell composition between control and UC groups. **(H)** Dot plot of biomarker expression in various cell types (where dot size and yellow intensity correspond to expression abundance). Significance markers: *p < 0.05, **p < 0.01, ***p < 0.001. ns, not significant.

To decipher the cellular atlas of UC, we analyzed two independent scRNA-seq datasets (GSE231993 and GSE125527). After rigorous quality control and batch effect correction, unsupervised clustering and annotation identified major cell populations, including Endothelial cells, T cells, B cells, and Macrophages (**Figures 10A, E**; **Supplementary Figures 2**-**3**). In both datasets, cell proportion analysis revealed significant disparities in myeloid lineage distributions between UC and control groups (**Figures 10B, C, F, G**). Further analysis of biomarker expression demonstrated that both *S100A8* and *RHOU* were highly expressed in macrophages across the GSE231993 dataset. This was partially validated in the GSE125527 cohort, where *S100A8* consistently exhibited the highest expression level in macrophage clusters; *RHOU* was not detected in the expression matrix, thus identifying macrophages as the primary cellular niche for these SGRG-associated biomarkers (**Figures 10D, H**).

### 3.7 Cell interaction and trajectory analyses reveal heterogeneity, metabolic reprogramming, and biomarker expression of macrophages in UC

**Figure 11:**
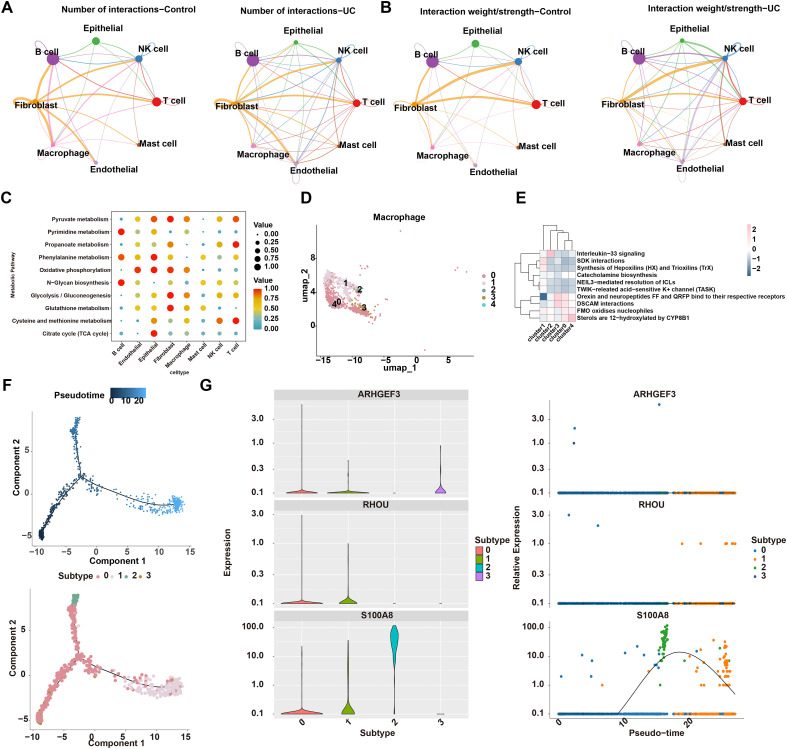
Changes in intercellular communication networks in ulcerative colitis (UC). **(A)** Number of annotated intercellular interaction networks in the UC and control groups. **(B)** Strength of annotated intercellular interaction networks in the UC and control groups. **(C)** Metabolic pathway analysis of each cell type. **(D)** Re-clustering of macrophages. **(E)** Enrichment analysis of macrophage subclusters. **(F)** Pseudotime analysis. **(G)** Expression changes of biomarkers during different macrophage differentiation stages. Different colors indicate distinct cellular subtypes and stages.

Cell communication analysis showed that as opposed to the control group, the count and intensity of connections between macrophages and other cell types (e.g., B cells, NK cells, T cells) were increased. Notably, the autologous communication of macrophages was absent (**Figures 11A, B**). Metabolic pathway analysis identified highly active pathways in macrophages, including “Pyruvate metabolism”, “Cysteine and methionine metabolism”, and “Oxidative phosphorylation” (**Figure 11C**). Macrophages were re-clustered into 5 subclusters after dimensionality reduction (dims = 10, resolution = 0.1). Specifically, subcluster 3 was significantly enriched in the DSCAM interactions pathway; subcluster 2 was significantly enriched in the Interleukin-33 signaling pathway; subcluster 0 was significantly enriched in FMO oxidizes nucleophiles; and subcluster 1 was significantly enriched in SDK interactions, etc. (**Figures 11D, E**). Trajectory analysis demonstrated that macrophages differentiated over time: subcluster 0 was mainly located in the early stage of differentiation, while subcluster 1 was predominantly in the late stage (**Figure 11F**). With cellular differentiation, the expression trend of *S100A8* first increased and then decreased throughout the differentiation process, whereas *ARHGEF3* and *RHOU* showed no significant changes (**Figure 11G**). These findings deepen our insight into the core role of macrophages in the etiopathogenesis of UC from multiple dimensions, including cell-cell interactions, metabolism, heterogeneity, and dynamic differentiation.

### 3.8 *In vivo* experimental validation of biomarkers at both mRNA and protein levels

**Figure 12:**
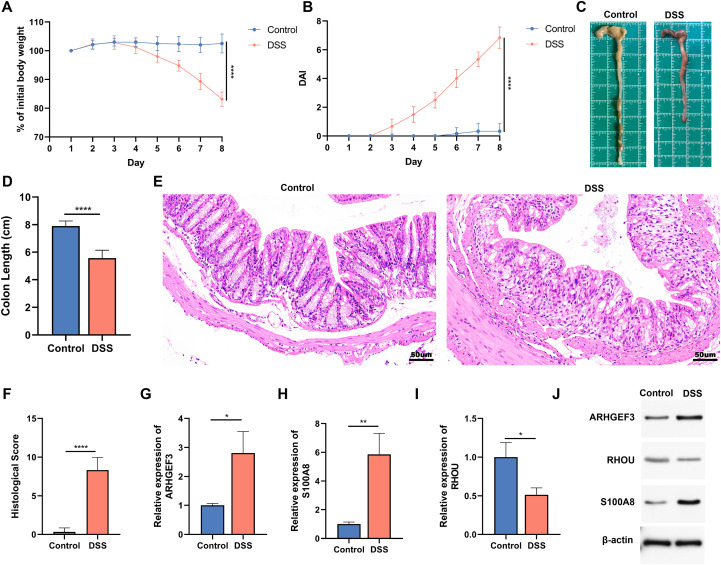
Establishment of the murine colitis model and validation of candidate biomarkers at both mRNA and protein levels. **(A, B)** Percentage body weight change and disease activity index (DAI) of mice in two groups. **(C)** Representative colon morphology and length of mice. **(D)** Quantitative analysis of colon length in mice. **(E)** Hematoxylin and eosin (H&E) staining of colon tissue sections in mice. Scale bar = 50 µm. **(F)** Histological scores of mice colon sections. **(G-I)** Relative expression of *ARHGEF3*, *S100A8*, and *RHOU* in colonic tissues of control mice and mice stimulated with dextran sulfate sodium (DSS). **(J)** Western blot analysis was performed to measure the levels of ARHGEF3, S100A8, and RHOU in mice colon tissues from different groups. Groups are identified as shown. Significance markers: *p < 0.05, **p < 0.01, ****p < 0.0001.

As indicated in the **Figures 12A–F**, we successfully established a mice experimental colitis model. To further explore the expression changes of core genes in the model, colon tissues from control mice and DSS-stimulated colitis mice were collected, and RNA was extracted for qRT-PCR analysis. Compared with the control mice, the expression of *ARHGEF3* and *S100A8* was significantly higher and *RHOU* significantly lower in the DSS-challenged mice (**Figures 12G–I**). To further corroborate these findings at the translational level, western blot analysis was performed. Consistent with the mRNA expression pattern, the protein levels of ARHGEF3 and S100A8 were markedly elevated in the colonic tissues of the DSS-induced colitis mice compared to the control mice, whereas the protein expression of RHOU was significantly reduced (**Figure 12J**). These results confirm that the identified biomarkers exhibit stable and consistent changes at both the transcriptional and translational levels during UC progression. These changes in gene expression are consistent with our bioinformatics results, providing important clues for further exploration of therapeutic targets for UC.

## 4 Discussion

UC is a chronic, nonspecific inflammatory disease of the intestine caused by multiple factors, commonly known as a major prevalent form of IBD (38). Studies have shown that the pathogenesis of UC is intimately associated with the destruction of the intestinal mucosal barrier and abnormal immune response (39). Meanwhile, as the key molecular switches of intracellular signal transduction, small GTPases profoundly affect the integrity of epithelial barrier by regulating cytoskeleton rearrangement, cell migration and tight junction assembly (40, 41). Therefore, based on multi-omics integration analysis, our study systematically reveals for the first time the potential of SGRGs (*ARHGEF3*, *S100A8*, and *RHOU*) as novel diagnostic biomarkers for UC. In the training set (GSE87466), *ARHGEF3* and *S100A8* were significantly upregulated in UC patients compared to the control group, whereas *RHOU* was significantly downregulated. These expression patterns were further confirmed in the external validation sets (GSE75214, GSE38713, and GSE107499), showing high consistency with the findings from the training set. Furthermore, all three nomograms consistently demonstrated that the identified biomarkers possessed high diagnostic value for UC. Beyond their diagnostic value, these biomarkers exhibited a significant correlation with UC disease severity, particularly *S100A8*, which showed a marked elevation during acute inflammatory flares. In addition, *ARHGEF3* displayed a unique capacity to differentiate UC from CD, likely reflecting the mucosal-centric epithelial barrier dysfunction specific to the UC phenotype. They were jointly enriched in pathways such as “Tight junction”, and were closely linked to the infiltration of immune cells including activated dendritic cells and effector memory CD8 T cells. ScRNA-seq further identified macrophages as key cells, and revealed enhanced communication between macrophages and other cells. It is particularly worth noting that *S100A8* exhibited a dynamic expression pattern during the differentiation process of macrophages. By integrating a broader range of transcriptomic data, the current study overcomes the potential bias associated with single-cohort analysis and provides stronger evidence for the involvement of SGRGs in UC pathogenesis. The consistent expression patterns of *ARHGEF3*, *S100A8*, and *RHOU* across multiple independent cohorts, coupled with the high AUC values and excellent calibration of the nomograms in external datasets, underscore the stability and reliability of these genes as potential clinical biomarkers for UC. These results not only advance the comprehension of the mechanism of small GTPases in UC, but also supply potential candidate targets for early diagnosis and precision therapy.

In terms of expression levels, *ARHGEF3* and *S100A8* were significantly upregulated in the UC group, whereas *RHOU* was significantly downregulated. As a member of the guanine nucleotide exchange factor for Rho GTPases, ARHGEF3 is a key activator of RhoGTPases, which is reported to mediate processes in platelet activation and cytoskeleton reorganization (42–44). The high expression of *ARHGEF3* in UC mucosa in our study indicates that it may drive the excessive activation of pathological RhoA/ROCK signaling. The activation of this pathway has been confirmed to directly disrupt epithelial tight junctions, increase barrier permeability, and promote immune cells migration. Therefore, the upregulation of *ARHGEF3* is very likely to be an important upstream event of epithelial barrier dysfunction and inflammatory infiltration in UC (45, 46). Similar molecular mechanisms have been reported in IBD models. For example, Rho kinase inhibitors have been proven to alleviate DSS-induced experimental colitis (47). The unique capacity of *ARHGEF3* to differentiate UC from CD may be attributed to the mucosal-restricted nature of UC pathology. CD involves transmural inflammation that penetrates all layers of the intestinal wall, while UC is characterized by inflammation primarily confined to the mucosal layer, where epithelial barrier failure serves as the central pathological event (3). Given that *ARHGEF3* is a pivotal regulator of the actin cytoskeleton and tight junction assembly, its significant dysregulation specifically in UC reflects this mucosal-centric disruption (42). Therefore, *ARHGEF3* serves as a biologically relevant marker that captures the essential molecular landscape of UC, distinguishing it from the deeper, more segmental tissue involvement in CD.

S100A8 is a principal effector of the inflammatory response (48), and its high expression in UC mucosa may be closely associated with inflammatory cell infiltration and tissue damage (49). Our results further confirmed the core position of *S100A8* in the UC immune microenvironment, and its expression level was strongly positively correlated with activated dendritic cells (cor = 0.94, p < 0.001). Dendritic cells have the functions of antigen presentation and immune activation (50). Hence, the upregulation of *S100A8* is an important link in the pathological chain that drives the abnormal activation of dendritic cells in the UC immune microenvironment and leads to the excessive amplification of subsequent T-cell immune responses. It is noteworthy that a recent study on osteoarthritis has shown that S100A8/A9 can drive monocytes to differentiate into M2-like macrophages and promote their phagocytic activity (51). Our single-cell analysis further characterized these dynamic changes during macrophage differentiation. While the pro-inflammatory influence of *S100A8* has been documented in general inflammatory models, its role in UC encompasses a distinct inflammatory and metabolic signature. Within the specific microenvironment of the colonic mucosa, S100A8 serves as a potent alarmin that binds to toll-like receptor 4 (TLR4) and receptor for advanced glycation end products (RAGE) on macrophages (52). Importantly, this interaction not only triggers NF-κB and MAPK-mediated cytokine production but also orchestrates fundamental metabolic reprogramming. S100A8-mediated TLR4 signaling is a known driver of the shift from oxidative phosphorylation toward aerobic glycolysis in macrophages. This glycolytic switch is essential for sustaining the rapid production of pro-inflammatory mediators, such as TNF-α and IL-1β, providing the necessary energy flux for cells to function within the hypoxic and nutrient-deprived environment of the inflamed colon (53–55). Immune metabolic reconfiguration is intrinsically coupled with the impairment of intestinal epithelial barrier. The synergy between S100A8-induced glycolytic activity and the hyper-secretion of cytokines likely exacerbates mucosal permeability by disrupting the “Tight junction” pathway identified in this study. This pathological impact is further corroborated by the existing evidence regarding S100A9, the primary binding partner of S100A8. Specifically, S100A9 has been shown to modulate the expression of tight junction proteins via the AMPK/mTOR signaling pathway, suggesting that the heterodimeric S100A8/A9 plays a synergistic role in barrier function regulation (56). The resulting vicious cycle—barrier disruption leading to antigen infiltration, antigen stimulation exacerbating inflammation, and inflammation further compromising the barrier—constitutes the pathological basis for the self-amplification of chronic tissue injury in UC. Furthermore, consistent with recent single-cell evidence, S100A8+ macrophages exhibit this non-classical polarization and metabolic pattern, acting as a major source of mucosal damage (49). This duality—acting as both an immune recruiter and a metabolic modulator—underlines its pivotal status in the pathological landscape of UC.

In contrast, RHOU is an atypical member of the Rho family of small GTPases and was significantly downregulated in UC (57). Previous studies have shown that *RHOU* is involved in the assembly of epithelial tight junctions, and the decline in its expression may lead to impaired epithelial barrier integrity (58), which is highly consistent with the enrichment results we observed in the “Tight junction” pathway. Additionally, *RHOU* was significantly negatively correlated with effector memory CD8 T cells (cor = -0.83, p < 0.001), reflecting that it may affect mucosal immune homeostasis by regulating T cell function (59). The heterogeneity in expression trend, that is, the upregulation of pro-inflammatory genes and the downregulation of barrier-related genes, reflects the complex interweaving of different molecular mechanisms in the pathological process of UC, and also suggests that both immunosuppression and epithelial repair need to be taken into account in treatment strategies.

In terms of immune infiltration, we evaluated the differences in 28 types of immune cells between the UC group and the control group using the ssGSEA algorithm, and found that neutrophils and activated dendritic cells were significantly increased in UC, which is consistent with previous studies related to UC immunity (60, 61). More importantly, the three key biomarkers showed a strong correlation with specific immune cell subsets, further supporting their potential role in immune regulation. For instance, *S100A8* is closely relevant to activated dendritic cells, and a study has confirmed that calprotectin composed of S100A8/A9 is a reliable biomarker for IBD, and its upregulation reflects the extensive activation of myeloid immune cells (such as activated dendritic cells), indicating that it may act as an amplifier of inflammatory responses (54, 62). The above suggests that *S100A8* is not only a passive indicator reflecting the degree of inflammation, but also likely to actively play a key role as an “amplifier of inflammatory response” in the pathological process. Furthermore, this study found a negative correlation between *RHOU* and effector memory CD8 T cells. Given that effector memory CD8 T cells are a key subpopulation driving mucosal inflammation in UC and inhibition of the Rho/ROCK signaling pathway can effectively alleviate T cell-mediated intestinal injury, the results indirectly provide evidence that the downregulation of *RHOU* expression may weaken the inhibition of mucosal injury mediated by effector memory CD8 T cells (63, 64). This study integrated bulk RNA sequencing and scRNA-seq data to comprehensively dissect the immune microenvironment of UC. Notably, there are certain differences between the two techniques in the assessment of cell abundance, which are mainly attributed to the following aspects: technical bias, such as the selective loss of fragile cells (e.g., neutrophils) easily caused by the tissue dissociation process in scRNA-seq; methodological differences, where deconvolution algorithms (e.g., ssGSEA) reflect population transcriptional activity (co-influenced by cell number and activity), while scRNA-seq provides physical cell counts; and cohort heterogeneity. Therefore, these two methods constitute complementary perspectives in this study: bulk data anchors broad clinical trends, while scRNA-seq provides a high-resolution cell state atlas, laying a reliable foundation for subsequent mechanism exploration.

ScRNA-seq further revealed the core position of macrophages in the UC microenvironment. We observed that macrophages not only increased in number in UC samples, but also exhibited significant metabolic reprogramming and functional heterogeneity. Macrophages with high expression of *S100A8* tend to differentiate into pro-inflammatory phenotypes, accompanied by the activation of mitochondrial pathways, which echoes the recent research on the role of “immunometabolism” in IBD (65). It is particularly worth noting that the recent study by Feng has demonstrated that CHI3L1 can promote pyroptosis of macrophages via the BCAT1/NF-κB axis, thereby exacerbating the progression of UC (66). Based on this, we speculate that *S100A8* may regulate macrophages through a pathway similar to that of CHI3L1 (such as NF-κB), thereby affecting the severity of UC. Moreover, we observed that *S100A8* expression showed dynamic changes with the differentiation status of macrophages, which was consistent with the conclusion reported by van Kooten, and further revealed the direct association between this biomarker and the core pathogenic cells of UC at the single-cell level (51). The central role of macrophages in UC pathogenesis, particularly the elevated expression of GTPase-associated biomarkers, was consistently observed across different scRNA-seq cohorts (GSE231993 and GSE125527). This cross-dataset validation provides strong evidence that macrophages serve as a primary cellular niche for SGRG-mediated regulation in the inflammatory microenvironment, regardless of cohort-specific variations.

This study is the first to systematically reveal the core role of *ARHGEF3*, *S100A8*, and *RHOU* as SGRGs in UC. Subsequently, the potential pathogenic mechanisms of SGRGs were further explored through methods such as immune cell infiltration analysis and GSEA, providing new directions for subsequent targeted interventions. However, this study also has some limitations. First, the currently proposed gene-pathway-phenotypic associations are mainly based on computational prediction and literature support. Second, the selected sample size is relatively small, and there may be deviations. The exact molecular mechanism still needs to be confirmed by subsequent experimental evidence. In the further, we will elucidate how *ARHGEF3*, *S100A8* and *RHOU* influence intestinal barrier integrity and immune cell function through molecular biological techniques involving their expression modulation (overexpression/knockdown).

## 5 Conclusion

This study is the first to systematically reveal the core role of *ARHGEF3*, *S100A8*, and *RHOU* as SGRGs in UC. It provides an important theoretical basis and experimental foundation for the screening of early diagnostic markers and the evaluation of disease prognosis in UC, and fills the relevant gaps in the research on the role of small GTPases family genes in the pathogenesis of UC.

## Data Availability

The datasets presented in this study can be found in online repositories. The names of the repository/repositories and accession number(s) can be found below: https://www.ncbi.nlm.nih.gov/, GSE87466, GSE75214, GSE231993, GSE38713, GSE107499, GSE11223, and GSE125527.

## References

[1] Zhang Y Ge F Qu H Zhao C Gu J Xu Q . Wumei Wan ameliorates ulcerative colitis in rats by modulating the inflammation-pyroptosis-intestinal stem cell axis. J Ethnopharmacol. (2026) 355:120645. doi: 10.1016/j.jep.2025.120645. PMID: 41005471

[2] Lian Z Hu J Cheng C Liu Y Zhu L Shen H . Association with controlling nutritional status score and disease activity of ulcerative colitis. J Int Med Res. (2023) 51:3000605231184046. doi: 10.1177/03000605231184046. PMID: 37548189 PMC10408351

[3] Le Berre C Honap S Peyrin-Biroulet L . Ulcerative colitis. Lancet. (2023) 402:571–84. doi: 10.1016/S0140-6736(23)00966-2. PMID: 37573077

[4] Du L Ha C . Epidemiology and pathogenesis of ulcerative colitis. Gastroenterol Clin North Am. (2020) 49:643–54. doi: 10.1016/j.gtc.2020.07.005. PMID: 33121686

[5] Zhou Q Shen ZF Wu BS Xu CB He ZQ Chen T . Risk of colorectal cancer in ulcerative colitis patients: A systematic review and meta-analysis. Gastroenterol Res Pract. (2019) 2019:5363261. doi: 10.1155/2019/5363261. PMID: 31781191 PMC6874962

[6] Singh S Murad MH Fumery M Dulai PS Sandborn WJ . First- and second-line pharmacotherapies for patients with moderate to severely active ulcerative colitis: An updated network meta-analysis. Clin Gastroenterol Hepatol. (2020) 18:2179–2191.e6. doi: 10.1016/j.cgh.2020.01.008. PMID: 31945470 PMC8022894

[7] Chen M Wei S Wu X Xiang Z Li X He H . 2’-Hydroxycinnamaldehyde alleviates intestinal inflammation by attenuating intestinal mucosal barrier damage via directly inhibiting STAT3. Inflamm Bowel Dis. (2024) 30:992–1008. doi: 10.1093/ibd/izad283. PMID: 38422244 PMC11144992

[8] Abbas A Di Fonzo DMP Wetwittayakhlang P Al-Jabri R Lakatos PL Bessissow T . Management of ulcerative colitis: where are we at and where are we heading? Expert Rev Gastroenterol Hepatol. (2024) 18:567–74. doi: 10.1080/17474124.2024.2422370. PMID: 39470444

[9] Li Y He X Xu C Wang R Shen G Li W . RhoA, a small GTPase, inhibits bacterial infection through regulated phagocytosis in the Chinese mitten crab, Eriocheir sinensis. Fish Shellfish Immunol. (2025) 167:110696. doi: 10.1016/j.fsi.2025.110696. PMID: 40907626

[10] Yang S Tang X Wang L Ni C Wu Y Zhou L . Targeting TLR2/Rac1/cdc42/JNK pathway to reveal that ruxolitinib promotes thrombocytopoiesis. Int J Mol Sci. (2022) 23:16137. doi: 10.3390/ijms232416137. PMID: 36555781 PMC9787584

[11] Paillares E Deboosere N Descorps-Declere S Marechal M Gillet D Demangel C . Screening of gene function in cell intoxication by CNF1 links Sec61 translocon to Rac1 GTPase activity. mBio. (2025) 16:e0258524. doi: 10.1128/mbio.02585-24. PMID: 41051206 PMC12607883

[12] Carangelo G Magi A Semeraro R . From multitude to singularity: An up-to-date overview of scRNA-seq data generation and analysis. Front Genet. (2022) 13:994069. doi: 10.3389/fgene.2022.994069. PMID: 36263428 PMC9575985

[13] Zheng HB . Application of single-cell omics in inflammatory bowel disease. World J Gastroenterol. (2023) 29:4397–404. doi: 10.3748/wjg.v29.i28.4397. PMID: 37576705 PMC10415967

[14] Qian C Hu C Xu Y Xu W Wang Z Gan W . Intestinal epithelial-derived USP13 alleviates colonic inflammation by suppressing GRP78-mediated endoplasmic reticulum stress. Adv Sci (Weinh). (2025) 12:e00741. doi: 10.1002/advs.202500741. PMID: 40679368 PMC12520541

[15] Du J Zhang J Wang L Wang X Zhao Y Lu J . Selective oxidative protection leads to tissue topological changes orchestrated by macrophage during ulcerative colitis. Nat Commun. (2023) 14:3675. doi: 10.1038/s41467-023-39173-2. PMID: 37344477 PMC10284839

[16] Xu M Zhou H Hu P Pan Y Wang S Liu L . Identification and validation of immune and oxidative stress-related diagnostic markers for diabetic nephropathy by WGCNA and machine learning. Front Immunol. (2023) 14:1084531. doi: 10.3389/fimmu.2023.1084531. PMID: 36911691 PMC9992203

[17] Wang J Wu N Feng X Liang Y Huang M Li W . PROS1 shapes the immune-suppressive tumor microenvironment and predicts poor prognosis in glioma. Front Immunol. (2022) 13:1052692. doi: 10.3389/fimmu.2022.1052692. PMID: 36685506 PMC9845921

[18] Zhang X Chao P Zhang L Xu L Cui X Wang S . Single-cell RNA and transcriptome sequencing profiles identify immune-associated key genes in the development of diabetic kidney disease. Front Immunol. (2023) 14:1030198. doi: 10.3389/fimmu.2023.1030198. PMID: 37063851 PMC10091903

[19] Song S Huang L Zhou X Yu J . Exploring the toxicological network in diabetic microvascular disease. Int J Surg. (2025) 111:3895–907. doi: 10.1097/js9.0000000000002394. PMID: 40214234 PMC12165556

[20] Wu T Hu E Xu S Chen M Guo P Dai Z . clusterProfiler 4.0: A universal enrichment tool for interpreting omics data. Innovation (Camb). (2021) 2:100141. doi: 10.1016/j.xinn.2021.100141. PMID: 34557778 PMC8454663

[21] Gu Z Gu L Eils R Schlesner M Brors B . circlize Implements and enhances circular visualization in R. Bioinformatics. (2014) 30:2811–2. doi: 10.1093/bioinformatics/btu393. PMID: 24930139

[22] Simon N Friedman J Hastie T Tibshirani R . Regularization paths for Cox’s proportional hazards model via coordinate descent. J Stat Softw. (2011) 39:1–13. doi: 10.18637/jss.v039.i05. PMID: 27065756 PMC4824408

[23] Yang L He S Tang L Qin X Zheng Y . Exploring immune-inflammation markers in psoriasis prediction using advanced machine learning algorithms. Front Immunol. (2025) 16:1619490. doi: 10.3389/fimmu.2025.1619490. PMID: 40821804 PMC12350470

[24] Hu J Szymczak S . A review on longitudinal data analysis with random forest. Brief Bioinform. (2023) 24:bbad002. doi: 10.1093/bib/bbad002. PMID: 36653905 PMC10025446

[25] Maurya NS Kushwah S Kushwaha S Chawade A Mani A . Prognostic model development for classification of colorectal adenocarcinoma by using machine learning model based on feature selection technique boruta. Sci Rep. (2023) 13:6413. doi: 10.1038/s41598-023-33327-4. PMID: 37076536 PMC10115869

[26] Robin X Turck N Hainard A Tiberti N Lisacek F Sanchez JC . pROC: an open-source package for R and S+ to analyze and compare ROC curves. BMC Bioinf. (2011) 12:77. doi: 10.1186/1471-2105-12-77. PMID: 21414208 PMC3068975

[27] Xu R Han F Zhao Y Liu A An N Wang B . Role of CENPL, DARS2, and PAICS in determining the prognosis of patients with lung adenocarcinoma. Transl Lung Cancer Res. (2024) 13:2729–45. doi: 10.21037/tlcr-24-696. PMID: 39507047 PMC11535832

[28] Feng R Cheng D Chen X Yang L Wu H . Identification and validation of palmitoylation metabolism-related signature for liver hepatocellular carcinoma. Biochem Biophys Res Commun. (2024) 692:149325. doi: 10.1016/j.bbrc.2023.149325. PMID: 38056161

[29] Liu C He Y Luo J . Application of chest CT imaging feature model in distinguishing squamous cell carcinoma and adenocarcinoma of the lung. Cancer Manag Res. (2024) 16:547–57. doi: 10.2147/cmar.S462951. PMID: 38855330 PMC11162187

[30] Revelle W Condon DM . Reliability from α to ω: A tutorial. Psychol Assess. (2019) 31:1395–411. doi: 10.1037/pas0000754. PMID: 31380696

[31] Hänzelmann S Castelo R Guinney J . GSVA: gene set variation analysis for microarray and RNA-seq data. BMC Bioinf. (2013) 14:7. doi: 10.1186/1471-2105-14-7. PMID: 23323831 PMC3618321

[32] Boland BS He Z Tsai MS Olvera JG Omilusik KD Duong HG . Heterogeneity and clonal relationships of adaptive immune cells in ulcerative colitis revealed by single-cell analyses. Sci Immunol. (2020) 5:eabb4432. doi: 10.1126/sciimmunol.abb4432. PMID: 32826341 PMC7733868

[33] Slovin S Carissimo A Panariello F Grimaldi A Bouché V Gambardella G . Single-cell RNA sequencing analysis: A step-by-step overview. Methods Mol Biol. (2021) 2284:343–65. doi: 10.1007/978-1-0716-1307-8_19. PMID: 33835452

[34] Darden DB Dong X Brusko MA Kelly L Fenner B Rincon JC . A novel single cell RNA-seq analysis of non-myeloid circulating cells in late sepsis. Front Immunol. (2021) 12:696536. doi: 10.3389/fimmu.2021.696536. PMID: 34484194 PMC8415415

[35] Li T Zhang W Niu M Wu Y Deng X Zhou J . STING agonist inflames the cervical cancer immune microenvironment and overcomes anti-PD-1 therapy resistance. Front Immunol. (2024) 15:1342647. doi: 10.3389/fimmu.2024.1342647. PMID: 38550593 PMC10972971

[36] Qing J Li C Zhi H Zhang L Wu J Li Y . Exploring macrophage heterogeneity in IgA nephropathy: Mechanisms of renal impairment and current therapeutic targets. Int Immunopharmacol. (2024) 140:112748. doi: 10.1016/j.intimp.2024.112748. PMID: 39106714

[37] Griss J Viteri G Sidiropoulos K Nguyen V Fabregat A Hermjakob H . ReactomeGSA - efficient multi-omics comparative pathway analysis. Mol Cell Proteomics. (2020) 19:2115–25. doi: 10.1074/mcp.TIR120.002155. PMID: 32907876 PMC7710148

[38] Li D Feng Y Tian M Ji J Hu X Chen F . Gut microbiota-derived inosine from dietary barley leaf supplementation attenuates colitis through PPARγ signaling activation. Microbiome. (2021) 9:83. doi: 10.1186/s40168-021-01028-7. PMID: 33820558 PMC8022418

[39] Pan HH Zhou XX Ma YY Pan WS Zhao F Yu MS . Resveratrol alleviates intestinal mucosal barrier dysfunction in dextran sulfate sodium-induced colitis mice by enhancing autophagy. World J Gastroenterol. (2020) 26:4945–59. doi: 10.3748/wjg.v26.i33.4945. PMID: 32952341 PMC7476174

[40] Faulkner B He Y Sitrin D Stains CI . Methods for controlling small GTPase activity. ChemBioChem. (2025) 26:e202500156. doi: 10.1002/cbic.202500156. PMID: 40396811 PMC12247031

[41] Tong J Wang Y Chang B Zhang D Wang B . Evidence for the involvement of RhoA signaling in the ethanol-induced increase in intestinal epithelial barrier permeability. Int J Mol Sci. (2013) 14:3946–60. doi: 10.3390/ijms14023946. PMID: 23429187 PMC3588079

[42] Khaliq SA Umair Z Yoon MS . Role of ARHGEF3 as a GEF and mTORC2 regulator. Front Cell Dev Biol. (2021) 9:806258. doi: 10.3389/fcell.2021.806258. PMID: 35174167 PMC8841341

[43] Ngwa JS Yanek LR Kammers K Kanchan K Taub MA Scharpf RB . Secondary analyses for genome-wide association studies using expression quantitative trait loci. Genet Epidemiol. (2022) 46:170–81. doi: 10.1002/gepi.22448. PMID: 35312098 PMC9086181

[44] Zou S Teixeira AM Kostadima M Astle WJ Radhakrishnan A Simon LM . SNP in human ARHGEF3 promoter is associated with DNase hypersensitivity, transcript level and platelet function, and Arhgef3 KO mice have increased mean platelet volume. PLoS One. (2017) 12:e0178095. doi: 10.1371/journal.pone.0178095. PMID: 28542600 PMC5441597

[45] Mihaescu A Santén S Jeppsson B Thorlacius H . Rho kinase signalling mediates radiation-induced inflammation and intestinal barrier dysfunction. Br J Surg. (2011) 98:124–31. doi: 10.1002/bjs.7279. PMID: 20882561

[46] Zou Y Ma L Zhao Y Zhang S Zhou C Cai Y . Inhibition of Rho kinase protects against colitis in mice by attenuating intestinal epithelial barrier dysfunction via MLC and the NF-κB pathway. Int J Mol Med. (2018) 41:430–8. doi: 10.3892/ijmm.2017.3197. PMID: 29115372

[47] López-Posadas R Becker C Günther C Tenzer S Amann K Billmeier U . Rho-A prenylation and signaling link epithelial homeostasis to intestinal inflammation. J Clin Invest. (2016) 126:611–26. doi: 10.1172/jci80997. PMID: 26752649 PMC4731169

[48] Ha JS Choi HR Kim IS Kim EA Cho SW Yang SJ . Hypoxia-induced S100A8 expression activates microglial inflammation and promotes neuronal apoptosis. Int J Mol Sci. (2021) 22:1205. doi: 10.3390/ijms22031205. PMID: 33530496 PMC7866104

[49] Li Y Wang Y Chen S Liu L . Dissecting macrophage heterogeneity in ulcerative colitis: Single-cell analysis and functional validation of S100A4 as a therapeutic target. Int Immunopharmacol. (2026) 168:115819. doi: 10.1016/j.intimp.2025.115819. PMID: 41242266

[50] Jin F Xie L Zhang H Fan X Tian J Liu W . Dendritic cells: Origin, classification, development, biological functions, and therapeutic potential. MedComm (2020). (2025) 6:e70455. doi: 10.1002/mco2.70455. PMID: 41200280 PMC12587171

[51] van Kooten NJT Blom AB Teunissen van Manen IJ Theeuwes WF Roth J Gorris MAJ . S100A8/A9 drives monocytes towards M2-like macrophage differentiation and associates with M2-like macrophages in osteoarthritic synovium. Rheumatol (Oxford). (2025) 64:332–43. doi: 10.1093/rheumatology/keae020. PMID: 38216750 PMC11701306

[52] Cho E Mun SJ Kim HK Ham YS Gil WJ Yang CS . Colon-targeted S100A8/A9-specific peptide systems ameliorate colitis and colitis-associated colorectal cancer in mouse models. Acta Pharmacol Sin. (2024) 45:581–93. doi: 10.1038/s41401-023-01188-2. PMID: 38040838 PMC10834475

[53] O’Neill LA Kishton RJ Rathmell J . A guide to immunometabolism for immunologists. Nat Rev Immunol. (2016) 16:553–65. doi: 10.1038/nri.2016.70. PMID: 27396447 PMC5001910

[54] Wang S Song R Wang Z Jing Z Wang S Ma J . S100A8/A9 in inflammation. Front Immunol. (2018) 9:1298. doi: 10.3389/fimmu.2018.01298. PMID: 29942307 PMC6004386

[55] de Oliveira Formiga R Li Q Zhao Y Campos Ribeiro MA Guarino-Vignon P Fatouh R . Immunometabolic reprogramming of macrophages by gut microbiota-derived cadaverine controls colon inflammation. Cell Host Microbe. (2025) 33:1855–1872.e10. doi: 10.1016/j.chom.2025.09.009. PMID: 41033313

[56] Chen C Sun B Chen K Bao H Tao Y Zhou J . Atractylenolide-I restore intestinal barrier function by targeting the S100A9/AMPK/mTOR signaling pathway. Front Pharmacol. (2025) 16:1530109. doi: 10.3389/fphar.2025.1530109. PMID: 40196359 PMC11973269

[57] Clayton NS Hodge RG Infante E Alibhai D Zhou F Ridley AJ . RhoU forms homo-oligomers to regulate cellular responses. J Cell Sci. (2024) 137:jcs261645. doi: 10.1242/jcs.261645. PMID: 38180080 PMC10917059

[58] Brady DC Alan JK Madigan JP Fanning AS Cox AD . The transforming Rho family GTPase Wrch-1 disrupts epithelial cell tight junctions and epithelial morphogenesis. Mol Cell Biol. (2009) 29:1035–49. doi: 10.1128/mcb.00336-08. PMID: 19064640 PMC2643799

[59] Zaric M Becker PD Hervouet C Kalcheva P Doszpoly A Blattman N . Skin immunisation activates an innate lymphoid cell-monocyte axis regulating CD8(+) effector recruitment to mucosal tissues. Nat Commun. (2019) 10:2214. doi: 10.1038/s41467-019-09969-2. PMID: 31101810 PMC6525176

[60] Espaillat MP Kew RR Obeid LM . Sphingolipids in neutrophil function and inflammatory responses: Mechanisms and implications for intestinal immunity and inflammation in ulcerative colitis. Adv Biol Regul. (2017) 63:140–55. doi: 10.1016/j.jbior.2016.11.001. PMID: 27866974 PMC5292058

[61] Ikeda Y Akbar F Matsui H Onji M . Characterization of antigen-presenting dendritic cells in the peripheral blood and colonic mucosa of patients with ulcerative colitis. Eur J Gastroenterol Hepatol. (2001) 13:841–50. doi: 10.1097/00042737-200107000-00013. PMID: 11474315

[62] Majster M Almer S Malmqvist S Johannsen A Lira-Junior R Boström EA . Salivary calprotectin and neutrophils in inflammatory bowel disease in relation to oral diseases. Oral Dis. (2025) 31:286–97. doi: 10.1111/odi.15036. PMID: 38852161 PMC11808171

[63] Casalegno Garduño R Däbritz J . New insights on CD8(+) T cells in inflammatory bowel disease and therapeutic approaches. Front Immunol. (2021) 12:738762. doi: 10.3389/fimmu.2021.738762. PMID: 34707610 PMC8542854

[64] Recaldin T Steinacher L Gjeta B Harter MF Adam L Kromer K . Human organoids with an autologous tissue-resident immune compartment. Nature. (2024) 633:165–73. doi: 10.1038/s41586-024-07791-5. PMID: 39143209 PMC11374719

[65] Li J He M Wan S Wang S Liu N Xin L . Engineering macrophage via biomaterial-mediated mitochondrial regulation: Mechanisms and strategies. Res (Wash D C). (2025) 8:883. doi: 10.34133/research.0883. PMID: 41262353 PMC12623607

[66] Feng J Zhang H Zhu M Zhang Q Rong H Shi T . CHI3L1 promotes macrophage pyroptosis in ulcerative colitis via the BCAT1/NF-κB axis. Life Sci. (2026) 384:124108. doi: 10.1016/j.lfs.2025.124108. PMID: 41314595

