# Supplementary material for: Integrated transcriptome and single-cell RNA sequencing identifies small GTPase-associated biomarkers in ulcerative colitis
Source: Front Immunol. 2026 Apr 22;17:1782885. doi: 10.3389/fimmu.2026.1782885 (PMC13143932; doi:10.3389/fimmu.2026.1782885)
Supplement: Supplementary file 10 [file DataSheet1.docx]

**Supplementary Method**

## Subcellular and chromosomal localization, functional similarity, and GeneMANIA analysis of biomarkers

To determine the specific cellular locations of the biomarkers, their subcellular localization data were retrieved from the Genecards database (https://www.genecards.org/). To clarify the positions of the biomarkers on human chromosomes, the RCircos package (v1.2.2) (1) was used to visualize their chromosomal distribution. Subsequently, functional similarity analysis was carried out with the GOSemSim package (v2.24.0) (2). Finally, the biomarkers were submitted onto the GeneMANIA online database (http://www.genemania.org) with the species parameter set to "Homo sapiens", and an interaction network involving the biomarkers and their functionally associated genes was constructed.

**Supplementary Results**

## Subcellular and chromosomal localization of biomarkers and interaction network analysis

Subcellular localization assay demonstrated that *ARHGEF3* was predominantly distributed in the cytoplasmic matrix, nucleus, and other cellular compartments; *RHOU* was relatively highly expressed in the cytoplasmic matrix, Golgi apparatus, and other structures; and *S100A8* was abundant in the cytoplasmic matrix, extracellular space, plasma membrane, and other locations (**Supplementary Figure 1A**). Chromosomal localization indicated that *ARHGEF3*, *RHOU*, and *S100A8* were mapped to chromosomes 3, 1, and 1, respectively (**Supplementary Figure 1B**). The similarity scores of the three biomarkers were all around 0.5, suggesting weak functional similarity among the genes (**Supplementary Figure 1C**). GeneMANIA predicted a total of 20 functionally related genes interacting with the biomarkers, including *S100A9*, *PAK1*, *S100A12*, etc. Co-expression network analysis using GeneMANIA indicated that there were 7 different types of interactions among these genes: physical interaction, co-expression, predicted interaction, etc. (**Supplementary Figure 1D**).

## ScRNA-seq data processing and analysis

The initial single-cell dataset (GSE231993) was utilized to decipher the cellular atlas and calculate the physical proportions of various cell types. While the cell frequency in scRNA-seq analysis (GSE231993) showed a distinct pattern from the bulk-derived infiltration scores (GSE87466), both datasets provided complementary evidence for the involvement of myeloid lineages. In the bulk analysis, the pivotal status of myeloid cell populations was evidenced by the exceptional correlation (cor = 0.94) between the myeloid-derived alarmin S100A8 and activated dendritic cells. This was further validated by scRNA-seq, which precisely localized *S100A8* expression to a specific pro-inflammatory macrophage cluster. The initial single-cell dataset (GSE231993) contained 39904 cells and 22471 genes. After quality control, a total of 26531 high-quality cells and 22471 genes were retained for subsequent analysis (**Supplementary Figure 2A**). Following data normalization, 2000 HVGs were extracted, with the top 10 including *IGHG2*, *IGHM*, *PYY*, *ZG16*, *TFF3*, *IGLC3*, *IGLC1*, *GCG*, *GUCA2A*, and *GUCA2B* (**Supplementary Figure 2B**). PCA and initial visualization revealed the presence of technical batch effects among different samples. Following the application of the Harmony algorithm, cells from different batches were observed to be well-intermingled in the reduced dimensional space. This integration ensures that the subsequent unsupervised clustering reflects true biological heterogeneity rather than technical artifacts, thereby satisfying the requirements for scientific reproducibility. The top 30 principal components contributed significant biological signals (p < 0.05) (**Supplementary Figure 2C**). Further clustering divided the cells into 21 cell clusters (resolution = 0.4) (**Supplementary Figure 2D**). The expression patterns of specific marker genes for these subpopulations were visualized via bubble plots (**Supplementary Figure 2E**).

To validate the key role of macrophages and to assess the generalizability of these cellular signatures across independent cohorts, we employed a second single-cell dataset (GSE125527) to decipher the cellular atlas and calculate the physical proportions of various cell types. The initial single-cell dataset (GSE125527) contained 32154 cells and 10105 genes. After quality control, a total of 30587 cells and 10105 genes were retained for subsequent analysis (**Supplementary Figure 3A**). Following data normalization, 2000 HVGs were extracted, identify the top 10 genes (**Supplementary Figure 3B**). Following the application of the Harmony algorithm, cells from different batches were observed to be well-intermingled in the reduced dimensional space. The top 25 principal components contributed significant biological signals (p < 0.05) (**Supplementary Figure 3C**). Further clustering divided the cells into 15 cell clusters (resolution = 0.5) (**Supplementary Figure 3D**). The expression patterns of specific marker genes for these subpopulations were visualized via bubble plots (**Supplementary Figure 3E**).

**References**

1. Zhang H, Meltzer P, Davis S. RCircos: an R package for Circos 2D track plots. *BMC Bioinformatics*. (2013) 14:244. doi: 10.1186/1471-2105-14-244

2. Yu G. Gene Ontology Semantic Similarity Analysis Using GOSemSim. *Methods Mol Biol*. (2020) 2117:207-15. doi: 10.1007/978-1-0716-0301-7_11


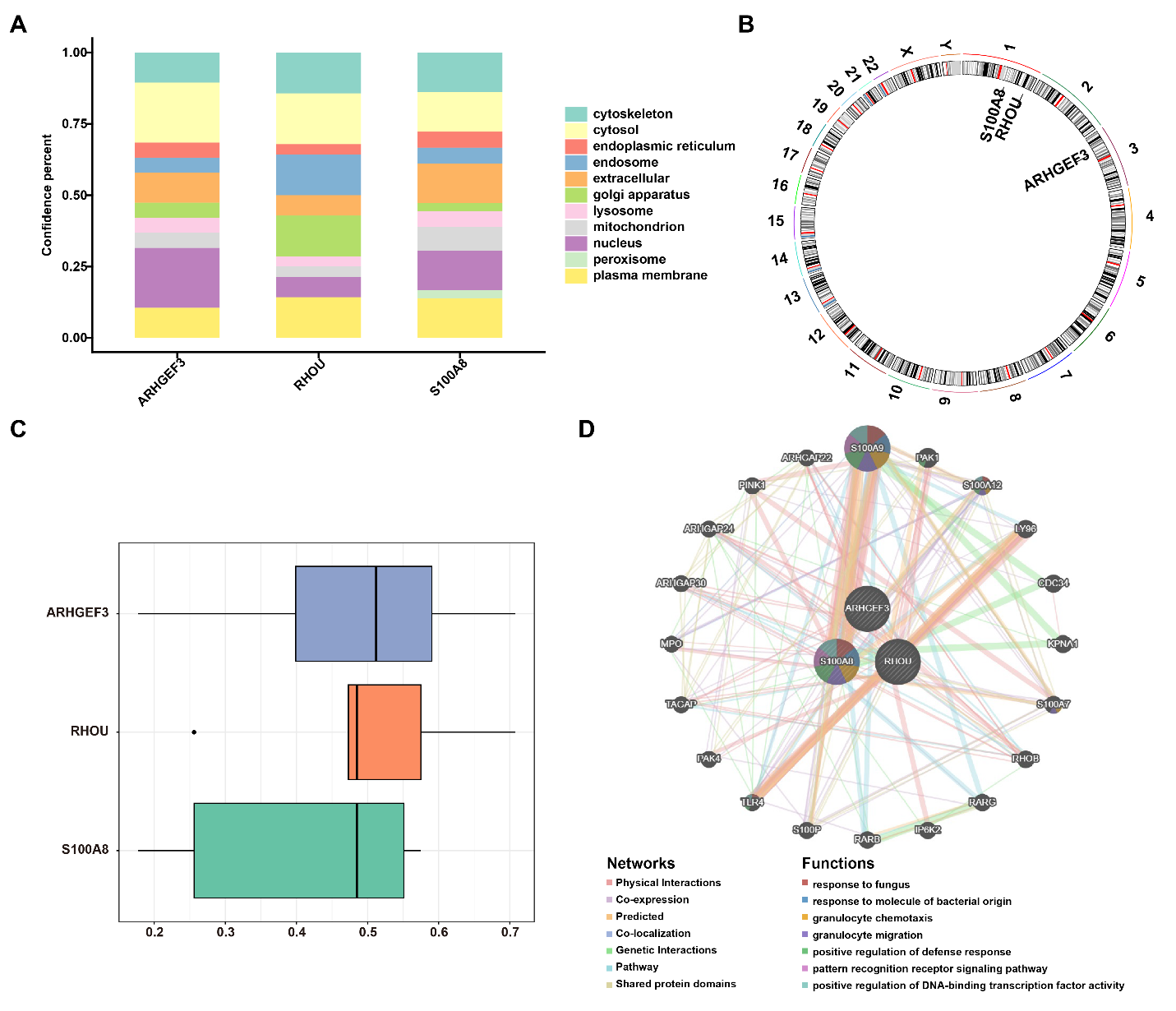


**Supplementary Figure 1** Subcellular and chromosomal localization of biomarkers and interaction network analysis. (**A**) Subcellular localization analysis. (**B**) Chromosomal localization analysis. (**C**) Functional similarity analysis. (**D**) GeneMANIA analysis.

**
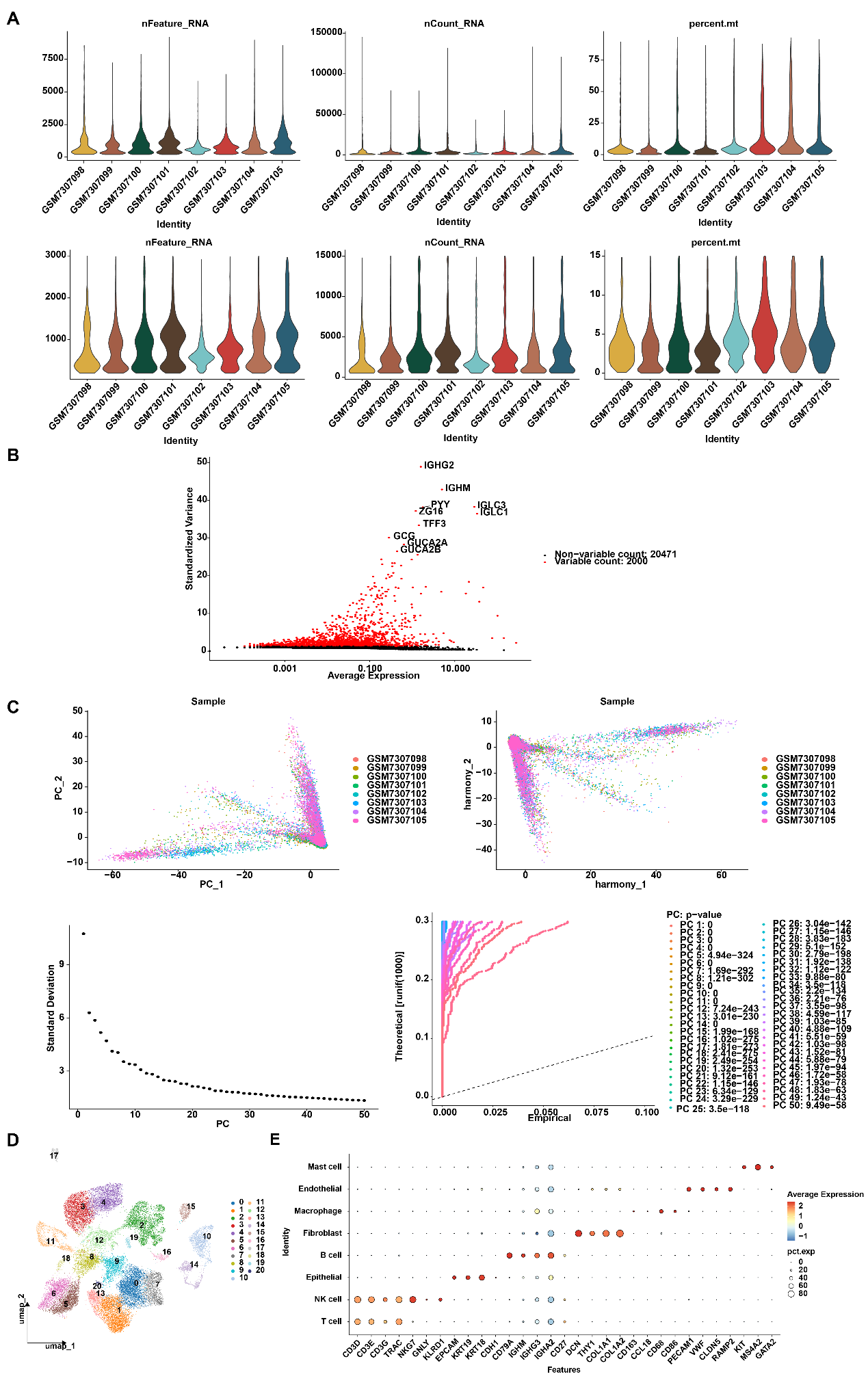
**

**Supplementary Figure 2** Single-cell RNA sequencing (ScRNA-seq) data processing and analysis (GSE231993 dataset). (**A**) Quality control results of single-cell data before and after filtering. nFeature_RNA = number of genes, nCount_RNA = number of cells, percent.mt = mitochondrial gene ratio; the x-axis shows sample information. (**B**) Screening results of highly variable genes (HVGs). (**C**) Principal component analysis (PCA) plots of cell distribution before and after batch effect correction, along with the scree plot and elbow plot. (**D**) Uniform manifold approximation and projection (UMAP) plot of cell clustering. (**E**) Expression of marker genes in cell clusters.


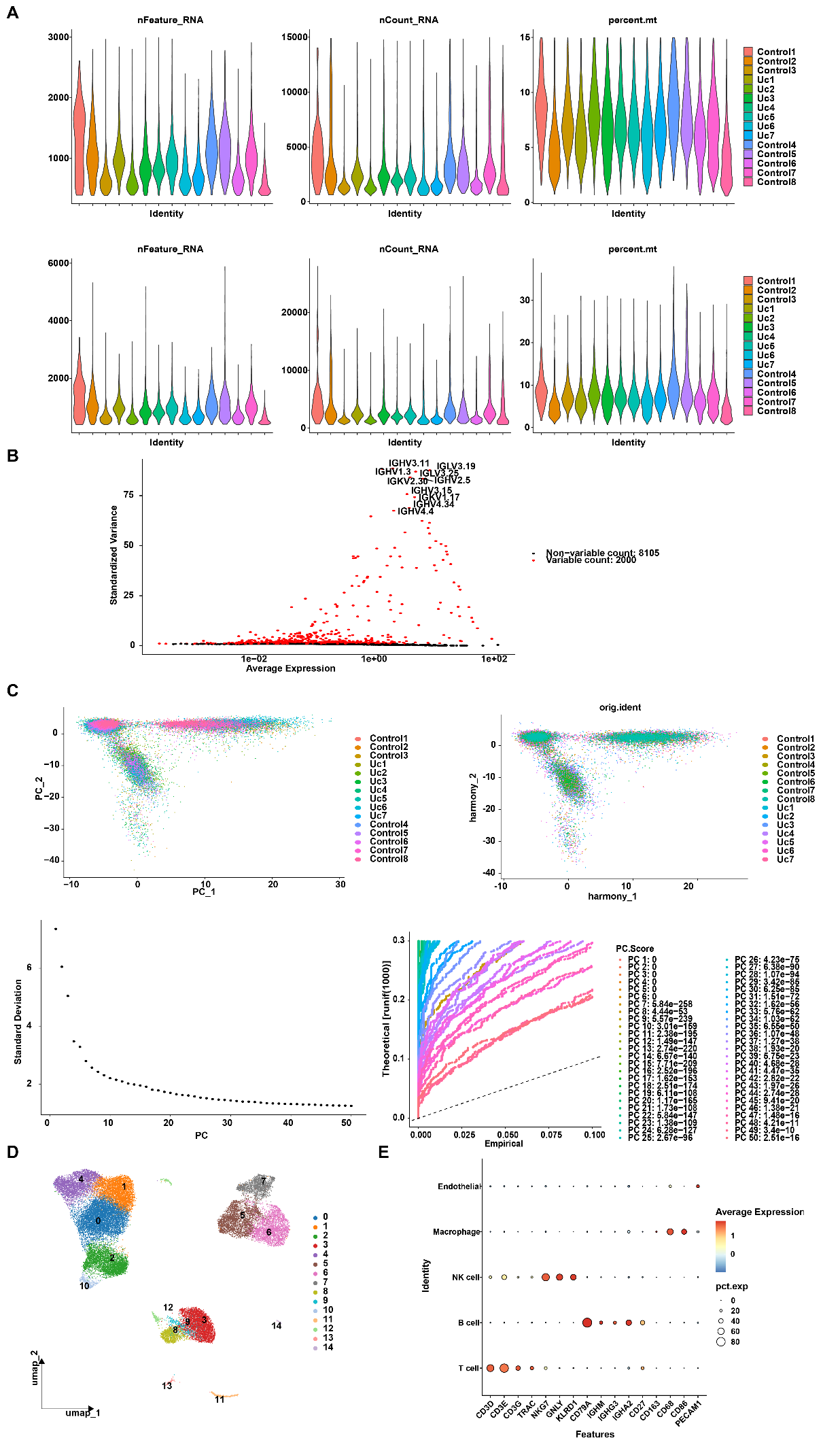
**Supplementary Figure 3** Single-cell RNA sequencing (ScRNA-seq) data processing and analysis (GSE125527 dataset). (**A**) Quality control results of single-cell data before and after filtering. nFeature_RNA = number of genes, nCount_RNA = number of cells, percent.mt = mitochondrial gene ratio; the x-axis shows sample information. (**B**) Screening results of highly variable genes (HVGs). (**C**) Principal component analysis (PCA) plots of cell distribution before and after batch effect correction, along with the scree plot and elbow plot. (**D**) Uniform manifold approximation and projection (UMAP) plot of cell clustering. (**E**) Expression of marker genes in cell clusters.
